# Coronin 1 Regulates Cognition and Behavior through Modulation of cAMP/Protein Kinase A Signaling

**DOI:** 10.1371/journal.pbio.1001820

**Published:** 2014-03-25

**Authors:** Rajesh Jayachandran, Xiaolong Liu, Somdeb BoseDasgupta, Philipp Müller, Chun-Lei Zhang, Despina Moshous, Vera Studer, Jacques Schneider, Christel Genoud, Catherine Fossoud, Frédéric Gambino, Malik Khelfaoui, Christian Müller, Deborah Bartholdi, Helene Rossez, Michael Stiess, Xander Houbaert, Rolf Jaussi, Daniel Frey, Richard A. Kammerer, Xavier Deupi, Jean-Pierre de Villartay, Andreas Lüthi, Yann Humeau, Jean Pieters

**Affiliations:** 1 Biozentrum, University of Basel, Basel, Switzerland; 2 Interdisciplinary Institute for Neuroscience, Bordeaux, France; 3 Hospital Necker, Paris, France; 4 Department of Radiology, University Children Hospital, UKBB, Basel, Switzerland; 5 Center for Cellular Imaging and NanoAnalytics, University of Basel, Basel, Switzerland; 6 Centre Hospitalier Universitaire de Nice, Nice, France; 7 Friedrich Miescher Institute, Basel, Switzerland; 8 University Hospital Basel, Basel, Switzerland; 9 Biomolecular Research Laboratory, Paul Scherrer Institute, Villigen, Switzerland; 10 Condensed Matter Theory, Paul Scherrer Institute, Villigen, Switzerland; University of Pennsylvania, United States of America

## Abstract

Cognitive and behavioral disorders are thought to be a result of neuronal dysfunction, but the underlying molecular defects remain largely unknown. An important signaling pathway involved in the regulation of neuronal function is the cyclic AMP/Protein kinase A pathway. We here show an essential role for coronin 1, which is encoded in a genomic region associated with neurobehavioral dysfunction, in the modulation of cyclic AMP/PKA signaling. We found that coronin 1 is specifically expressed in excitatory but not inhibitory neurons and that coronin 1 deficiency results in loss of excitatory synapses and severe neurobehavioral disabilities, including reduced anxiety, social deficits, increased aggression, and learning defects. Electrophysiological analysis of excitatory synaptic transmission in amygdala revealed that coronin 1 was essential for cyclic–AMP–protein kinase A–dependent presynaptic plasticity. We further show that upon cell surface stimulation, coronin 1 interacted with the G protein subtype Gαs to stimulate the cAMP/PKA pathway. The absence of coronin 1 or expression of coronin 1 mutants unable to interact with Gαs resulted in a marked reduction in cAMP signaling. Strikingly, synaptic plasticity and behavioral defects of coronin 1–deficient mice were restored by *in vivo* infusion of a membrane-permeable cAMP analogue. Together these results identify coronin 1 as being important for cognition and behavior through its activity in promoting cAMP/PKA-dependent synaptic plasticity and may open novel avenues for the dissection of signal transduction pathways involved in neurobehavioral processes.

## Abstract 2

The evolutionarily conserved protein coronin 1 is needed for activating the cyclic AMP signaling pathway in the brain and is important for cognition and behavior.

## Author Summary

Memory and behavior depend on the proper transduction of signals in the brain, but the underlying molecular mechanisms remain largely unknown. Coronin 1 is a member of a highly conserved family of proteins, and although its gene lies in a chromosome region associated with neurobehavioral dysfunction in mice and men, it has never been directly ascribed a specific function in the brain. Here we show that coronin 1 plays an important role in cognition and behavior by regulating the cyclic AMP (cAMP) signaling pathway. We find that when cell surface receptors are activated, coronin 1 stimulates cAMP production and activation of protein kinase A. Coronin 1 deficiency resulted in severe functional defects at excitatory synapses. Furthermore, in both mice and humans, deletion or mutation of coronin 1 causes severe neurobehavioral defects, including social deficits, increased aggression, and learning disabilities. Strikingly, treatment with a membrane-permeable analogue of cAMP restored synaptic plasticity and behavioral defects in mice lacking coronin 1. Together this work not only shows a critical role for coronin 1 in neurobehavior but also defines a role for the coronin family in regulating the transmission of signals within cells.

## Introduction

Behavioral and cognitive deficits comprise a heterogeneous collection of pathologies. Copy number variants and several single gene alterations predisposing to neurobehavioral and cognitive diseases have been identified and are believed to act either independently or in a combinatorial fashion [1],[2]. The molecular functions of the candidate genes that are associated with cognitive and behavioral impairment are beginning to be elucidated [1]; several of these molecules were shown to be located at synapses, suggesting that synaptic dysfunction is involved in neurobehavioral disorders [3]–[6]. However, for many of the candidate genes a direct link with neurobehavioral disorders as well an understanding of their molecular function remains unknown [7],[8].

An important neuronal signaling cascade involved in synaptic plasticity and learning occurs downstream of G protein–coupled receptors, resulting in the activation of adenylate cyclase that produces cAMP through stimulation with the Gαs subunit of trimeric G proteins [9]–[12]. cAMP in turn activates protein kinase A (PKA), which drives long-term changes in synaptic efficacy through direct effects on the pre- or postsynapse and through CREB-dependent regulation of gene transcription [13]–[15]. However, the mechanisms regulating cAMP production remain incompletely understood.

In this article we describe a crucial role for the conserved WD repeat protein coronin 1 in cognition and behavior through the activity of coronin 1 in modulating the cAMP/PKA pathway. Coronin 1 is encoded in a genomic region on chromosome 16 in human and the corresponding region of mouse chromosome 7 whose copy number variations are associated with varying degrees of cognitive impairment [16]–[19]. Coronin 1 is a member of the WD repeat containing protein family, and is expressed in immune cells as well as in nervous tissue [20]–[22]. In immune cells, coronin 1 is required for the transduction of cell surface signals to intracellular signaling cascades, thereby regulating a number of different processes, ranging from pathogen destruction to the survival of T cells [22]–[26]. A function for coronin 1 in neuronal cells or tissues has, however, not been described.

We here show that mice lacking coronin 1 displayed increased aggression, social deficits, increased repetitive behavior, reduced fear/anxiety, and a severe defect in learning and memory. We found that coronin 1 was specifically expressed in excitatory synapses and required for cAMP/PKA-dependent synaptic plasticity. We further show that upon cellular stimulation, coronin 1 interacts with the G protein subunit Gαs to stimulate the cAMP/PKA pathway. Importantly, infusion of the cAMP analogue 8-Br-cAMP reversed the learning and memory deficits in coronin 1–deficient mice. These results identify coronin 1 as being important for cognition and behavior through its activity in modulating cAMP/PKA-dependent synaptic plasticity.

## Results

### Coronin 1 Deficiency Results in Altered Behavior

**Figure 1:**
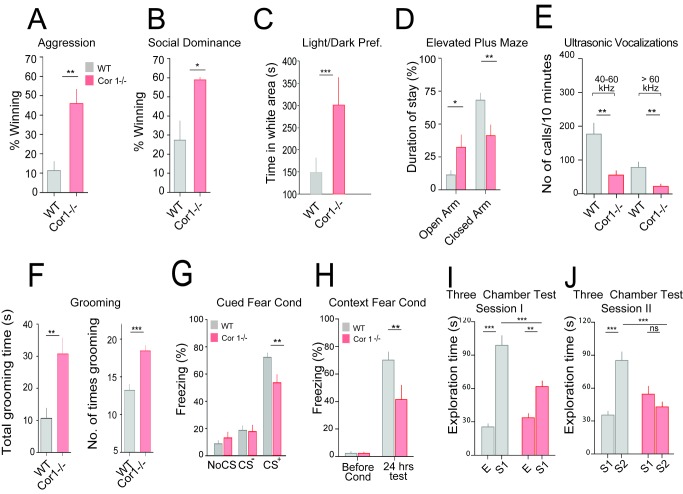
Increased aggression and impaired memory and socialization in the absence of coronin 1. (A) Resident intruder aggression analysis. The mouse that initiated the fight and displayed dominance was scored as the winner. *N* = 37 trials, *p*<0.01. (B) Tube displacement test for social dominance analysis. The first mouse to exit the tube was scored the loser and the other the winner (socially dominant). *N* = 33 trials, *p*<0.05. (C). Light versus dark preference. *N* = 14 WT and 16 coronin 1 −/−, *p*<0.001, Student's *t* test. (D) Elevated plus maze analysis (*n* = 16 wild-type and 15 coronin 1 −/−). Open arm, *p*<0.05; closed arm, *p* = 0.01. (E) The number of calls emitted in ultrasonic frequencies. *n* = 15 wild-type and 13 coronin 1 −/−, *p*<0.01 for 40–60 kHz and *p*<0.01 for >60 kHz, Student's *t* test. (F) For self-grooming, the duration (*p*<0.01, left) and number of times (*p*<0.0001, right) was scored. *n* = 13 per genotype. (G) Twenty-four hours after cued fear conditioning, coronin 1–deficient mice exhibit impaired long-term memory as indicated by reduced freezing levels during CS+ exposure. Wild-type, *n* = 12 and coronin 1 −/−, *n* = 11 mice. *p*<0.01, RM-ANOVA. Freezing during CS− exposure or in the absence of auditory stimulation was not different. (H) Mean contextual freezing during a 4 min context exposure before and 24 h after contextual fear conditioning. Twenty-four hours after contextual fear conditioning, coronin 1 −/− mice exhibit impaired long-term memory as indicated by reduced freezing levels compared to wild-type mice. *p*<0.01, RM-ANOVA. *N* = 5 WT and 6 coronin 1 −/− mice. (I, J) Three chamber analysis. Coronin 1 −/− mice show a reduced sociability in session 1 (I) and a reduced social novelty in session 2 (J) relative to wild-type mice. *n* = 16 wild-type and 16 coronin 1–deficient mice. **p<0.01 and ***p<0.001, RM-ANOVA, see Table S1 for additional statistics. Wild type: gray bars; coronin 1-deficient: red bars.

In the course of analyzing mice harboring a targeted deletion of the coronin 1 gene [23],[26] we consistently observed behavioral abnormalities. In particular, coronin 1–deficient mice showed a significantly enhanced aggressive behavior, shorter attack latency, and social dominance compared to wild-type mice (Figure 1A,B and Table S1). Also, upon transfer to a dark versus light chamber, mice lacking coronin 1 showed an increased duration of stay in the light compartment relative to wild-type as well as an increased number of transitions between the dark and light chambers and a reduced latency to enter the light compartment (Figure 1C and Figure S1A). Analysis using the elevated plus maze test revealed that coronin 1–deficient mice spent a significantly longer duration of time in the open arm of the maze (Figure 1D and Figure S1B). Furthermore, analysis of vocalization [27] revealed a reduction in calls in coronin 1–deficient mice compared to wild-type animals (Figure 1E). Finally, we analyzed self-grooming, which in mice is assumed to be an equivalent to stereotypic behavior observed in models of behavioral abnormalities [28],[29]. As shown in Figure 1F, coronin 1–deficient mice showed a drastically increased self-grooming. Together these results suggest that the absence of coronin 1 results in reduced anxiety and increased aggression.

### Coronin 1 Deficiency Results in Defective Memory Formation and Socialization

Analysis of classical cued fear conditioning [30] showed that coronin 1–deficient mice exhibited markedly reduced freezing levels as compared to wild-type animals (Figure 1G and Figure S1D,E). Likewise, contextual fear conditioning, induced by exposing mice to a single unconditioned stimulus (US) in a neutral environment, was strongly impaired in the absence of coronin 1 (Figure 1H), although no defects in locomotion or pain thresholds nor perception were observed as assessed by Y-maze, grip test, rotarod, beam walking, open field, formalin pain sensitivity, hot plate, and tail flick analysis (Figure S1C,E,F and Table S1), suggesting that the behavioral defects are not the result of low exploratory activity and/or impaired sensory abilities. Also, analysis of novel object recognition did not reveal any difference between wild-type and coronin 1–deficient mice (Figure S1G). Next, we monitored the degree of socialization of both wild-type and coronin 1–deficient mice using the three-chamber socialization test [27],[28]. Wild-type and coronin 1–deficient mice showed similar olfactory abilities (Figure S2A) and did not display any side preferences or differences in the number of entries into the chambers in the absence of a companion mouse (Figure S2B–E). However, coronin 1–deficient mice showed a significantly reduced sociability as well as reduced preference for social novelty (Figure 1I,J). A similar social defect was observed using a modified Paylor's partition analysis (Figure S2F), which, together with the three-chamber analysis, suggests defective social interaction upon coronin 1 deletion.

To analyze the consequences of coronin 1 depletion in human, we evaluated an individual from a consanguineous family presenting with immunodeficiency due to a homozygous missense mutation in the coronin 1 coding region, causing a valine to methionine change at the conserved amino acid position 134 (Figure S3A) that resulted in coronin 1 deficiency [31]. To rule out gene duplication or deletion of the 16p11.2 fragment, which is known to be associated with cognitive and behavioral dysfunction, we performed conventional karyotyping and high-resolution array CGH analysis that did not reveal genetic alterations (see Materials and Methods). Furthermore whole exome sequencing did not show any homozygous mutation in another gene that may be linked to neurocognitive impairment. Bioinformatic structural predictions revealed that this valine is located at a critical position in the interface between two blades [32],[33], tightly packed against Val106, His130, and Ser149. As a consequence the coronin 1^V134M^ mutant was rapidly degraded (Figure S3B,C). The neurological examination of the patient was normal, presenting no microcephaly, hypertonia, spasticity, or anomaly of cranial nerves and no facial dysmorphic features. Broad biological, metabolic, and medical screenings in order to rule out other causes for developmental delay were performed, with no abnormalities being diagnosed (see Text S1 and Table S2). However, neuropsychological examination revealed that although the patient acquired gross motor milestones timely (crawling at 8 months and free walking at 12 months, respectively), he presented a severe delay in language acquisition as well as significant behavioral anomalies including increased levels of aggression (scratching and biting), attention/concentration deficit, hyperactivity, impulsivity, and sleep disturbances (Figure S3D,E). Furthermore, in intelligence and achievement tests, the patient presented a significantly lower performance than his age-matched controls (Figure S3F). Together these observations indicate that loss of coronin 1 results in severe cognitive and behavioral dysfunction.

### Neuronal Coronin 1 Is Expressed in Excitatory But Not Inhibitory Neurons and Regulates the Excitatory/Inhibitory (E/I) Synapse Ratio

**Figure 2:**
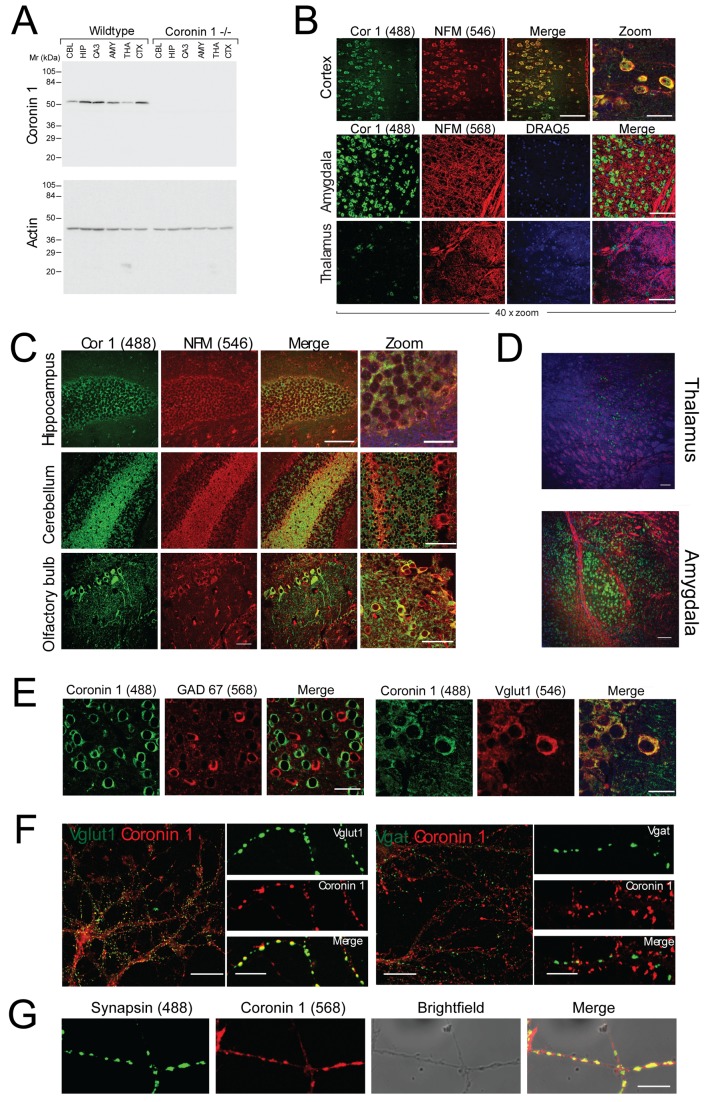
Coronin 1 distribution and localization in excitatory and inhibitory neurons. (A) Brain regions as indicated were dissected from wild-type or coronin 1–deficient mice, lysed, and immunoblotted for coronin 1 (upper panel) or actin (lower panel). (B) Sections from wild-type mouse brain were analyzed for the expression of coronin 1 (Alexa Fluor 488) and neurofilament-M chain (Alexa Fluor 546 and 568) as well as DRAQ5. Scale bar, 100 µm (Merge); 25 µm (Zoom). (C) Sections from wild-type mouse brain were analyzed for the expression of coronin 1 (Alexa Fluor 488) and neurofilament-M chain (Alexa Fluor 546). Scale bar, 100 µm (merge); 25 µm (zoom). (D) Sections from wild-type mouse brain were analyzed for the expression of coronin 1 (Alexa Fluor 488) and neurofilament-H (Alexa Fluor 546). 10× magnification and imaged with a confocal microscope (scale bar, 100 µm). (E) Sections from wild-type mouse brain (cortex) were labeled with anti-GAD67 (left panels) or anti-Vglut (right panels) as well as anti–coronin 1 antibodies, followed by Alex Fluor 568 or 546 as well as 488 labeled secondary antibodies. Scale bar, 25 µm (left) and 10 µM (right). (F) Hippocampal neurons were labeled using ani-coronin 1 antibodies and double labeled using either anti-vGLUT1 or anti-vGAT antibodies, followed by Alex Fluor 568 (for coronin 1) or 488 (for Vglut and Vgat) labeled secondary antibodies, respectively. (G) Hippocampal neurons isolated from wild-type mice were labeled using anti–synapsin 1 and anti–coronin 1 antibodies, followed by Alex Fluor 488 or 568 labeled secondary antibodies, respectively. Scale bar, 10 µm.

The above results suggest a function for coronin 1 in cognition and behavior. Analysis of neuronal distribution of coronin 1 by immunohistochemistry and immunoblotting revealed expression throughout different brain regions, including expression in cortex, basolateral amygdala, as well as olfactory bulb, hippocampus, cerebellar molecular layer, with minimal expression in the thalamus (Figure 2A–D and Figure S4). Notably, coronin 1 expression was found to be mutually exclusive with the expression of GAD67, a marker for GABAergic inhibitory neurons [34], and was predominantly found in neurons expressing the glutamate transporter vGLUT1 specific to excitatory neurons (Figure 2E) [35]. Consistent with the expression pattern found *in vivo*, in hippocampal neuronal cultures, coronin 1 colocalized with vGLUT1 but not with vGAT, markers of excitatory and inhibitory presynapses, respectively (Figure 2F). Coronin 1 was highly enriched in synapses, as judged by the extensive colocalization with synapsin 1 (Figure 2G) [36]. Together, these results suggest that coronin 1 is enriched at excitatory synapses.

**Figure 3:**
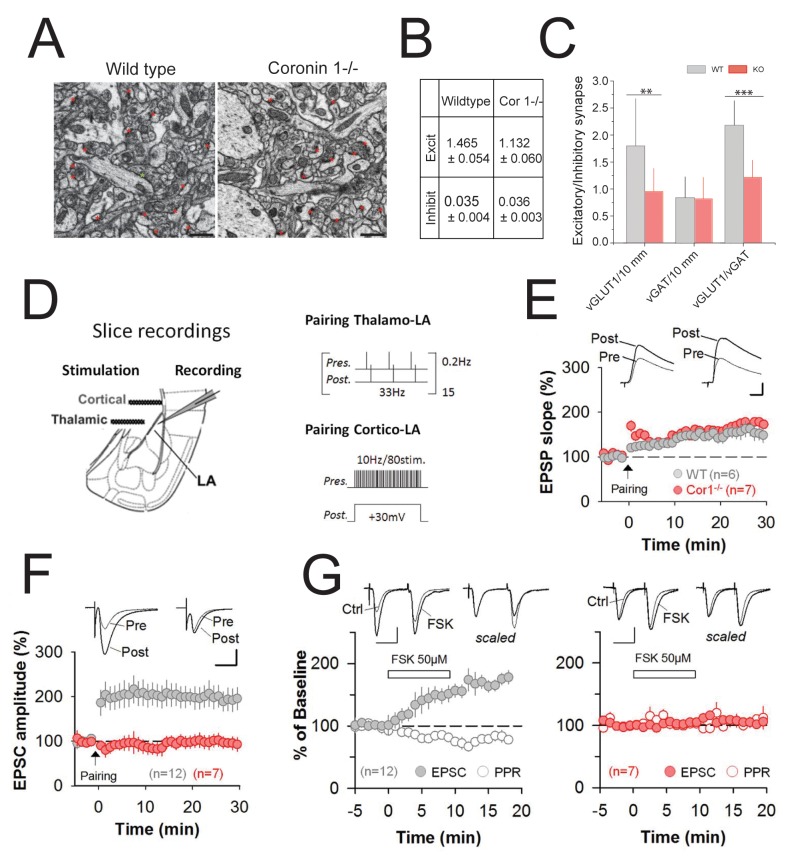
Decreased E/I synapse ratio and loss of cortico-LA synaptic plasticity in the absence of coronin 1. (A, B) Analysis of synapses in brain tissue from wild-type or coronin 1–deficient mice (*n* = 3) by serial block face scanning by electron microscopy. Scale bar, 2 microns. Data shown are mean values ± SD, *p*<0.01. (C) Hippocampal neurons were labeled using anti-vGLUT1 and anti-vGAT antibodies, followed by Alex Fluor 488 or 568 labeled secondary antibodies, respectively. The average number of inhibitory and excitatory synapses per 10 µm, as well as the E/I synapse ratio from wild-type or coronin 1–deficient neurons were determined. Quantitation (right panel) was done from 60 neuron terminals from four mice per genotype. For vGLUT1, *p*<0.01; for vGAT, *p*>0.05; and for vGLUT/vGAT ratio, *p*<0.001, Student's *t* test. (D) Scheme of the experimental preparation (LA, lateral amygdala) and pairing protocols used to induce LTP. (E) Coronin 1–deficient animals (Cor1 −/−, red symbols) exhibited normal thalamo-LA LTP [*p*<0.05 versus baseline; *p*>0.05 versus wild-type (WT, gray symbols), Student's *t* test]. Scale bars, 2 mV and 10 ms. (F) Cortico-LA LTP is completely absent in coronin 1–deficient mice (red symbols). Strong cortico-LA LTP was induced in wild-type [WT (grey symbols), *n* = 12, *p*<0.05 versus baseline, *p*<0.05 versus Cor 1 −/− (red symbols), Student's *t* test), but not in coronin 1–deficient mice [Cor 1 −/− (red), *n* = 7, *p*>0.05 versus baseline, Student's *t* test]. Scale bars, 50 pA and 20 ms. (G) Synaptic transmission and PPR in wild-type (left) and coronin 1–deficient mice (right) in the presence of forskolin. Forskolin enhances synaptic transmission and decreases PPR at cortico-LA synapses in WT (*n* = 12, *p*<0.05 versus pre-forskolin baseline), but not in Cor 1 −/− (*n* = 7, *p*>0.05 versus pre-forskolin baseline, Student's *t* test) mice. Scale bars, 100 pA and 20 ms. See Table S1 for additional statistics.

Cognitive and behavioral dysfunction has been linked to an imbalance of excitatory and inhibitory synapses [6],[37]. To analyze the consequences of coronin 1 deletion for the E/I synapse ratio, excitatory and inhibitory synapses in wild-type and coronin 1–deficient hippocampi were examined by serial block face scanning electron microscopy (see Materials and Methods). Strikingly, although the number of inhibitory synapses was not affected by coronin 1 deletion, the number of excitatory synapses was significantly reduced (Figure 3A,B), similar to the reduction of vGLUT1 but not vGAT synapses from in vitro cultured neurons (Figure 3C). In addition, electrophysiological analysis showed a significant reduction of the E/I miniature events frequency ratio in the absence of coronin 1 (Figure S5).

### Regulation of PKA-Dependent Synaptic Plasticity by Coronin 1

To assess the functional role of coronin 1 at excitatory synapses in anxiety-related brain regions, whole-cell current and voltage-clamp recordings were performed from lateral amygdala (LA) projection neurons, while stimulating excitatory pathways from cortex and thalamus, the two main sensory inputs onto LA principal neurons (Figure 3D). Baseline synaptic transmission as well as postsynaptic long-term potentiation (LTP) at thalamo-LA synapses [38] was found to be largely normal in the absence of coronin 1 (Figure 3E and Figure S6). However, PKA-dependent presynaptic cortico-LA LTP [39] was completely absent in coronin 1–deficient animals (Figure 3F). Next, to bypass upstream induction mechanisms, we applied forskolin, which directly increases the presynaptic probability of release at cortico-LA synapses [39]. Although in wild-type mice application of a brief pulse of forskolin persistently increased excitatory postsynaptic potential (EPSC) amplitude and decreased the paired pulsed ratio (PPR), forskolin had no effect on synaptic transmission in coronin 1–deficient mice (Figure 3G). We therefore conclude that the absence of cortico-LA LTP in coronin 1–deficient amygdala results from defective cAMP/PKA signaling.

**Figure 4:**
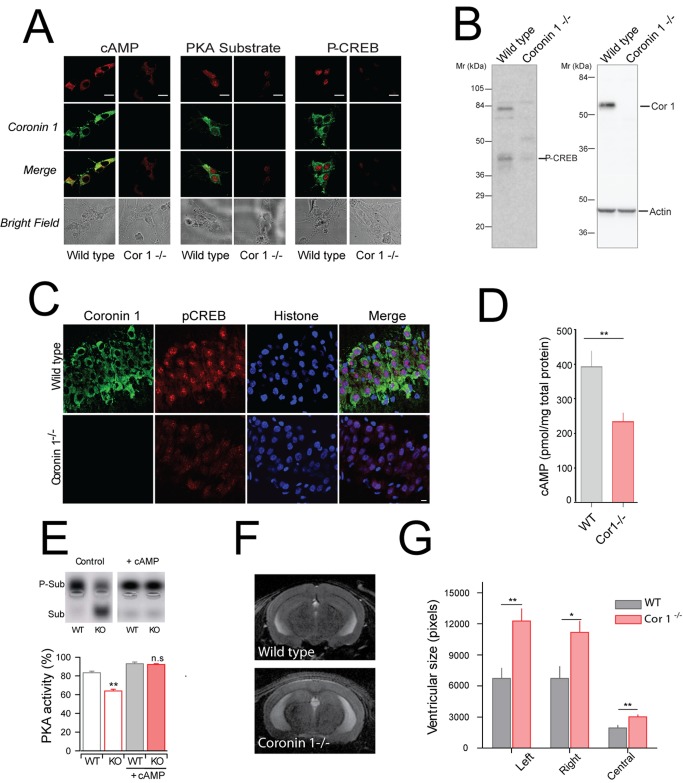
cAMP production, P-CREB analysis, and ventricle sizes in the presence and absence of coronin 1. (A) Hippocampal neurons (7 d culture) were fixed and stained with antibodies against coronin 1 (Alexa 488) and cAMP-, PKA substrate-, or P-CREB antibodies (568). Scale bar, 20 µm. (B) Wild-type or coronin 1–deficient brain lysates were immunoblotted using anti-P-CREB antibodies and reprobed using anti–coronin 1 and anti-actin antibodies. (C) Sections from wild-type or coronin 1–deficient brain were immunolabeled using anti–P-CREB antibodies, anti–coronin 1, and anti-histone antibodies. Scale bar, 10 µm. (D) Amygdalary regions of wild-type and coronin 1–deficient mice were analyzed for cAMP levels by ELISA. The values shown are normalized to total protein amounts (pmol cAMP/mg total protein). The data shown are from three independent experiments; *n* = 10 mice per genotype, *p*<0.01, Student's *t* test, see Table S1. (E) Basal (Control) and cAMP-stimulated PKA activity was determined in brain lysates from wild-type (WT) and coronin 1–deficient (KO) mice using a Peptag assay. PKA activity was monitored by the negative charge increase of the PKA substrate-containing peptide. (Upper panel) Electrophoretic pattern. (Lower panel) Quantitation (*n* = 3), *p* = 0.01. (F, G) Ventricle sizes in the presence and absence of coronin 1 as analyzed by MRI on live animals. H, mean ventricular size. *N* = 6, left *p*<0.01, right *p*<0.05, and middle *p*<0.01, Student's *t* test, see Table S1.

In neurons, as in other cells, PKA activation leads to the phosphorylation of the cAMP response element binding protein (CREB) at serine 133, thereby activating CREB, which is required for CREB-mediated gene transcription that is involved in the regulation of cognitive functioning, memory consolidation, and the late phase of postsynaptic LTP [14],[15],[40]. Staining of wild-type and coronin 1–deficient hippocampal neurons with antibodies directed against cAMP, a PKA consensus site, or phospho-CREB revealed strongly reduced labeling in coronin 1–deficient neurons (Figure 4A). Furthermore, analysis of brain lysates revealed significantly increased CREB phosphorylation at serine-133 only in the presence of coronin 1 (Figure 4B). Similarly, in brain sections, P-CREB labeling was severely reduced in neurons from coronin 1–deficient animals compared to wild-type (Figure 4C). Consistent with reduced cAMP levels in coronin 1–deficient mouse brain (Figure 4D), direct measurement of PKA activity in amygdala lysates showed a significantly reduced activity in the absence of coronin 1 compared to wild-type animals (Figure 4E). Inclusion of cAMP during the assay resulted in an elevation of the PKA activity in coronin 1–deficient brain sections comparable to that in wild-type sections, indicating that PKA is fully functional in the absence of coronin 1. A decrease in neuronal cAMP levels was recently found to be associated with increased ventricular size [41]. Interestingly, when analyzing brains from wild-type and coronin 1–deficient mice using *in vivo* magnetic resonance imaging (MRI), we observed that deletion of coronin 1 resulted in a significant enlargement of the lateral ventricles in adult but not in young mice (Figure 4F,G and Figure S7A,B), corroborating a role for neuronal coronin 1 in the modulation of cAMP signaling. Ventricle enlargement was associated with a reduction in the cell numbers in the hippocampus, whereas basic histopathology of brain sections did not reveal any other obvious differences (Figure S7A–C and unpublished data). Together these results suggest an essential role for coronin 1 in activating cAMP-dependent signaling, thereby impacting memory and behavior via modulating synaptic plasticity.

### Coronin 1–Dependent cAMP Production in Transfected Cells Analyzed by FRET

**Figure 5:**
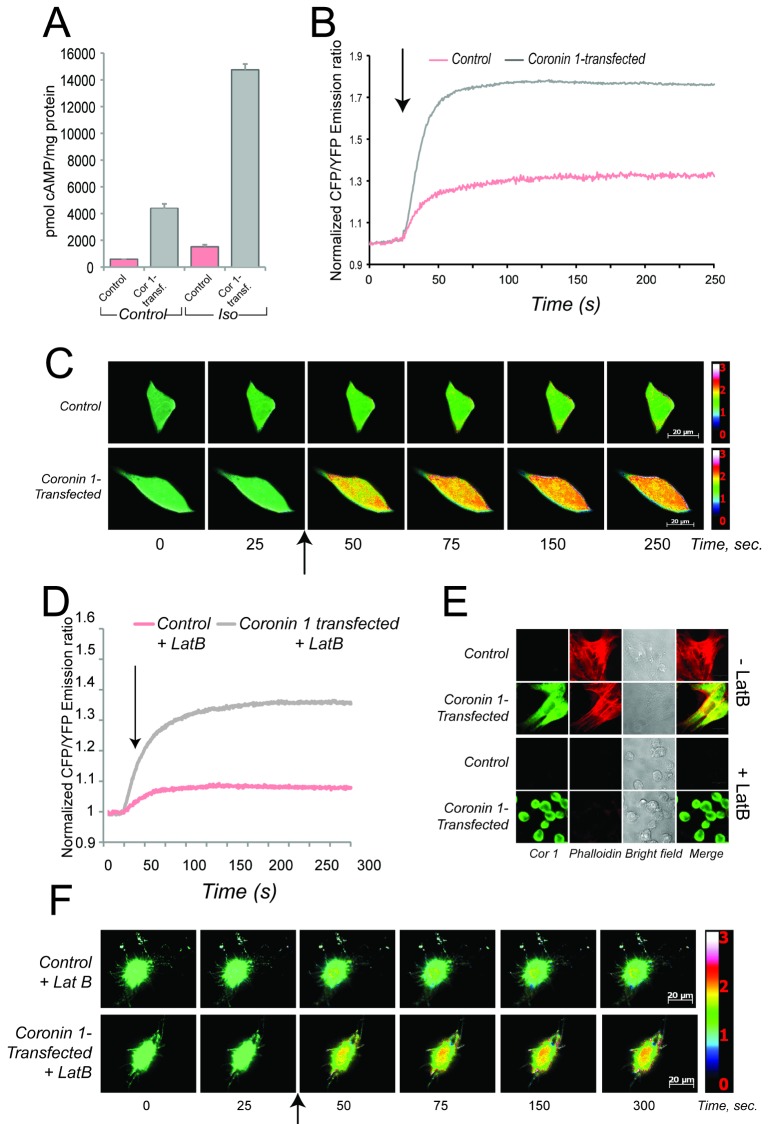
ELISA and FRET-based analysis of cAMP production in coronin 1–positive and coronin 1–negative cells in the presence and absence of F-actin. (A) Coronin 1–expressing or control Mel JuSo cells were left untreated or stimulated with isoproterenol (5 µM, 4 min) and processed for cAMP analysis using ELISA (see Materials and Methods). The values were normalized to total protein amounts (pmol cAMP/mg protein). The data shown are a representative of three independent experiments. Data represent mean and SEM. (B, C) FRET-based analysis of cAMP production upon stimulation of wild-type or coronin 1–expressing Mel JuSo cells that had been transfected with pICUE3 with isoproterenol (10 µM). (B) the normalized emission ratio (CFP/YFP) time course of coronin 1–expressing (gray) or wild type (red) Mel JuSo cells shown in C (mean, *n* = 50). (C) Stills from movies representing the normalized CFP/YFP emission ratios. Arrows indicate the time of isoproterenol addition. See also Movies S1 and S2. (D–F) FRET-based analysis of cAMP production upon stimulation of Latrunculin B–treated (4 µM, 30 min) wild-type or coronin 1–expressing Mel JuSo cells (transfected with pICUE3) with isoproterenol (5 µM, at T = 25 s). (D) The normalized emission ratio (CFP/YFP) time course of coronin 1–expressing (grey) or wild-type (red) Mel JuSo cells (mean, *n* = 30). (E) Coronin 1 and F-actin staining in untreated and Latrunculin B–treated cells. (F) Stills from movies representing the normalized CFP/YFP emission ratios. Arrows indicate the time of isoproterenol addition. See also Movies S3 and S4.

To address the molecular mechanism underlying the coronin 1–dependent activation of cAMP/PKA signaling, we analyzed stimulus-dependent cAMP production in a coronin 1–negative cell line expressing or lacking coronin 1 (Mel JuSo cells, see [20]). When these cells were left untreated or stimulated with isoproterenol, cAMP production, as analyzed using a competitive antibody assay, was drastically increased by the expression of coronin 1 (Figure 5A). Expression or deletion of coronin 1 did not influence β2-adrenergic receptor levels at the cell surface, nor did it modulate the expression of other molecules involved in cAMP production, including adenylate cyclases, β-adrenergic receptor, Gαs, and Gαi (see Figure S8).

To analyze coronin 1–dependent cAMP production in real time, we used a live cell cAMP reporter based on the activity of the exchange protein activated by cAMP 1 (Epac1), a guanine exchange factor for the small GTPase Rap1 (ICUE3) [42],[43]. Upon cAMP binding, the Epac1 fusion protein undergoes a conformational change that results in a decrease in fluorescence resonance energy transfer (FRET) efficiency, visualized as an increase in the CFP∶YFP ratio. Stimulation of coronin 1–expressing cells resulted in a ratiometric increase (CFP∶YFP) that originated at the plasma membrane, rapidly increasing and spreading over the entire cell body (Figure 5B,C and Movie S1). In contrast, in coronin 1–negative cells, a severe reduction in the ratiometric increase was observed (Figure 5B,C and Movie S2). Together these results corroborate the role for coronin 1 in the activation of cAMP signaling.

### Coronin 1–Mediated Activation of cAMP Signaling and the F-Actin Cytoskeleton

Coronin 1 colocalizes with the F-actin cytoskeleton in vitro as well as within cells. To directly test whether the coronin 1–mediated cAMP production was dependent on the F-actin cytoskeleton, cells expressing or lacking coronin 1 were treated with the F-actin depolymerizing drug latrunculin B [44] for 30 min, followed by stimulation with isoproterenol and analyzed by FRET. Although latrunculin B fully depolymerized the actin cytoskeleton, as evidenced by the absence of any F-actin staining (Figure 5E), the presence of latrunculin B did not affect coronin 1–dependent cAMP production as analyzed by FRET (Figure 5D,F and Movie S3 and Movie S4). Together these results suggest that coronin 1 promotes cAMP production in an F-actin–independent manner.

### Stimulus-Dependent Interaction of Coronin 1 with Gαs

**Figure 6:**
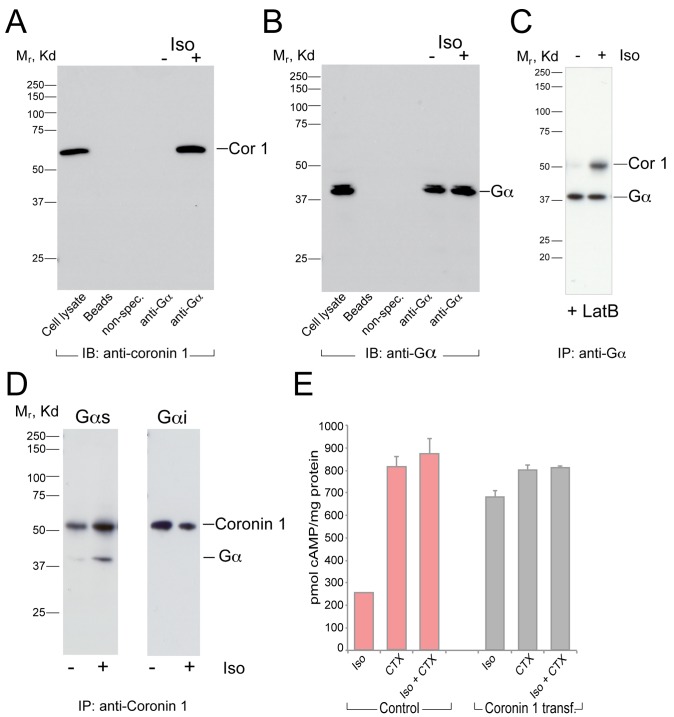
Stimulus-dependent association of coronin 1 with Gαs. (A, B) Coronin 1–expressing Mel JuSo cells were left untreated or stimulated with isoproterenol (10 µM, 10 min), lysed, and Gα molecules were immunoprecipitated with the indicated antibodies as described, followed by separation on SDS-PAGE and immunoblotting using anti–coronin 1 (A) or anti-Gα antibodies (B). The appearance of Gα as a doublet may be a result of posttranslational modifications and/or partial cleavage. (C) Coronin 1–expressing Mel JuSo cells were treated with Latrunculin B (4 µM, 30 min; see Figure 5D–F), lysed, and proteins immunoprecipitated using anti-Gα antibodies, followed by SDS-PAGE and immunoblotting. (D) NIE-115 cells were stimulated with isoproterenol (10 µM, 10 min), lysed, and immunoprecipitated using anti–coronin 1 antibodies. Protein complexes were separated by SDS-PAGE and immunoblotted for coronin 1 and the Gα molecules indicated. (E) Coronin 1–expressing Mel JuSo cells or control cells were starved in rolipram (100 µM, 1 h) and stimulated with cholera toxin (1 µg/mL) for 1 h at 37°C prior to stimulation with isoproterenol (10 µM, 4 min). cAMP production was measured as described in Materials and Methods.

The above results strongly suggest that upon stimulation with isoproterenol, coronin 1 functions in enhancing the production of cAMP that results from β2-adrenergic receptor stimulation. To analyze whether the coronin 1–dependent cAMP production resulted from a physical interaction between coronin 1 and Gαs, cells expressing coronin 1 were activated with isoproterenol, lysed, and Gα complexes immunoprecipitated using specific antibodies. Protein complexes were separated by SDS-PAGE and immunoblotted using either anti-Gα or anti–coronin 1 antibodies. Although only low to undetectable amounts of coronin 1 were observed in anti-Gα immunoprecipitates from unstimulated cells, upon isoproterenol stimulation the coronin 1 signal drastically increased (Figure 6A,B). In accordance with the capacity of coronin 1 to promote cAMP production in an F-actin–independent manner, depolymerization of F-actin by latrunculin B did not affect the stimulus-dependent interaction of coronin 1 with Gα (Figure 6C). Similarly, coronin 1 was co-immunoprecipitated with Gα in cell lysates from stimulated N1E-115 neuronal cells, whereas low to undetectable amounts of coronin 1 were co-immunoprecipitated with Gα in unstimulated cells (Figure S9). To analyze whether co-immunoprecipitated Gα molecules represented Gαs, as suggested by the lack of inhibition by pertussis toxin (Figure S10), coronin 1 was immunoprecipitated from untreated or isoproterenol-stimulated N1E-115 cells, and immunoblotted for either Gαs or Gαi. As shown in Figure 6D, although Gαs was readily detected in coronin 1 immunoprecipitates following isoproterenol stimulation, Gαi could not be detected. Furthermore, addition of cholera toxin, which permanently ADP ribosylates and thereby constitutively activates Gαs, induced an equivalent cAMP production in both coronin 1–expressing and coronin 1–deficient cells (Figure 6E).

**Figure 7:**
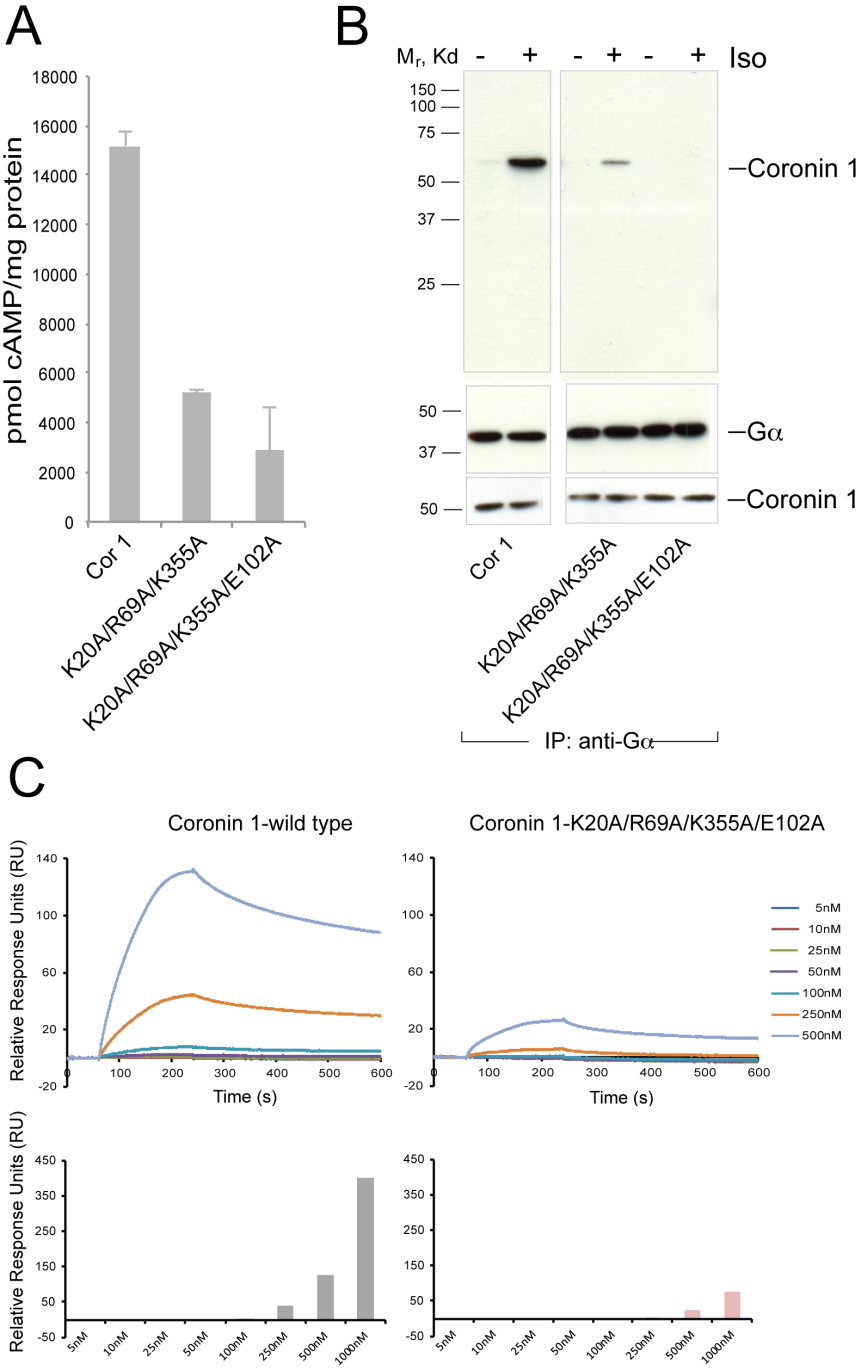
Analysis of coronin 1–Gα interaction in cells and in vitro by surface plasmon resonance. (A, B) Cells were transfected with the different constructs indicated, stimulated with 10 µM isoproterenol (A, 4 min; B, 10 min) followed by lysis and analyzed for cAMP. Panel B shows the results of immunoprecipitating Gα followed by immunoblotting for coronin 1 (upper panels). The lower panels in B show the immunoblots for Gα and coronin 1 following SDS-PAGE of the transfected cell lysates. (C) Purified Gαs was covalently immobilized on an NTA sensor chip surface through EDC/NHS chemistry. The indicated concentrations of coronin 1 or coronin 1 mutant were sequentially injected into the SPR sensor chip. The kinetic data were collected and analyzed. The Relative Response Units (RUs) of stable binding under each different concentration is shown in the lower panel.

Next, we investigated the possibility that the previously shown results could arise from a direct interaction between coronin 1 and Gα. Because the crystal structure of coronin 1 is known [33], we generated a series of coronin 1 mutant molecules in which residues at the surface of coronin 1 (and therefore potentially involved in protein–protein interactions within a complex) were mutated to alanine (see Table S3). Coronin 1 mutant molecules were expressed in Mel JuSo cells, and the capacity to stimulate cAMP production following stimulation of the β2-adrenergic receptor with isoproterenol was analyzed by cAMP ELISA. As shown in Figure 7A, mutation of Lys-20, Arg-69, Glu-102 alone or in combination with Lys-355 strongly decreased cAMP production. Importantly, these coronin 1 mutants, which were stably expressed and correctly localized (Figure 7B lower panels and Figure S11), were unable to associate with Gα (Figure 7B). In addition, the role of the mutated residues in the interaction with Gαs was assessed by surface plasmon resonance experiments between purified immobilized Gαs and either purified wild-type coronin 1 or the purified coronin 1 alanine quadruple mutant (coronin 1^K20A/R69A/K355A/E102A^). Strikingly, the interaction of the coronin 1 quadruple mutant with Gαs was drastically decreased as compared to the wild-type coronin 1 (Figure 7C). Together these data suggest that coronin 1 interacts with Gαs to promote cAMP production in a stimulus-dependent manner.

### Rescue of the Coronin 1–Dependent Learning Deficit by in Vivo 8-Br-cAMP Administration

**Figure 8:**
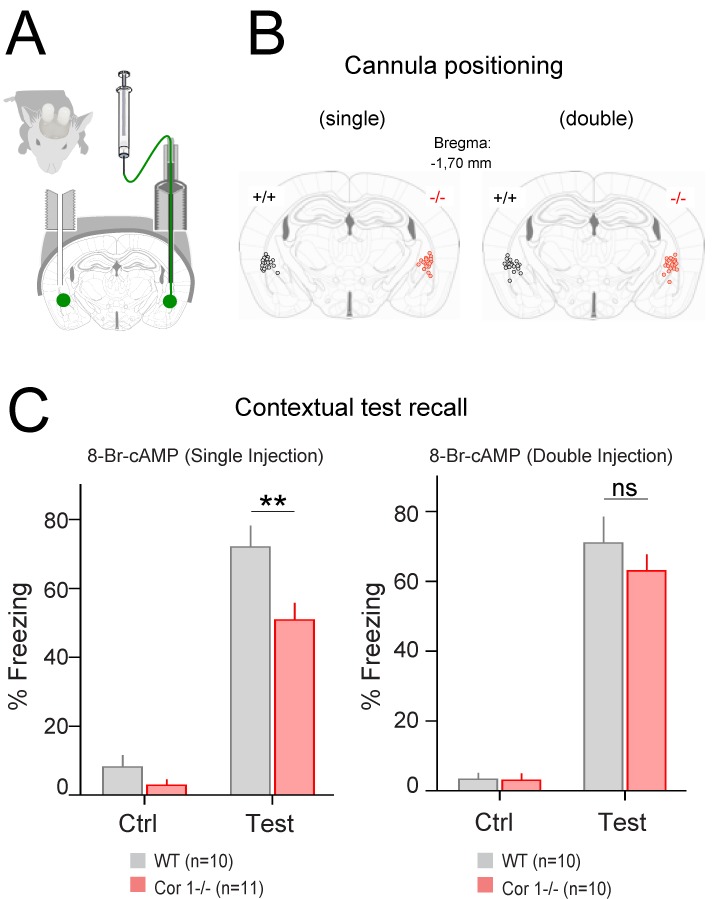
Rescue of fear conditioning in coronin 1–deficient mice by in vivo 8-Br-cAMP infusion into amygdala. (A) Scheme of the in vivo preparation. (B) Cannula implantation loci in both animal cohorts: single injection mice (left) and double injection mice (right). (C) Contextual learning was tested before (Ctrl) and 12 h later following single (*n* = 10 WT and 11 coronin 1 −/−) and double cAMP injections (*n* = 10 for both genotypes). Single injections, *p*<0.01. Double injections, *p*>0.05, RM-ANOVA, see also Table S1.

Together the data thus far reveal a role for coronin 1 in learning and memory via promoting cAMP-dependent plasticity through the association with Gαs. We next wanted to directly test whether the defective cAMP/PKA signaling associated with coronin 1 deficiency was responsible for the lack of fear consolidation as revealed by the low freezing levels observed during the recall tests (Figure 1G,H). To do so, we tested whether some of the behavioral deficits observed in coronin 1–deficient mice could be rescued by timely applications of the membrane-permeable nonhydrolysable cAMP analogue 8-Br-cAMP [45] within the amygdala (Figure 8). To that end, 8-Br-cAMP was bilaterally infused in the amygdala of wild-type and coronin 1–deficient animals via chronically implanted cannula (Figure 8A,B). To correct presynaptic plasticity during CS/US association, cAMP injections were performed 30∼45 min prior to fear acquisition in coronin 1–deficient animals. When tested *in vitro*, such 8-Br-cAMP pre-incubations partially rescued the absence of plasticity at cortico-LA synapses in coronin 1–deficient slices (Figure S12). Infusion of 8-Br-cAMP in amygdala failed to normalize fear reactions upon contextual re-exposure in coronin 1–deficient mice (Figure 8C “single injection”). As such a defect might be due to a lack of CREB activation in the hours following neuronal activation [46], we performed an additional cAMP infusion 2.5∼3 h after the fear acquisition. Importantly, this second drug application was able to restore the contextual fear recall defect in coronin 1–deficient animals, which became indistinguishable from similarly treated wild-type animals (Figure 8C “double injection”). Together with the rescued LTP in coronin 1–deficient slices by application of 8-Br-cAMP, these pharmacological results obtained *in vivo* strongly support a role for coronin 1 in learning and memory through the activation of cAMP-dependent synaptic plasticity.

## Discussion

Cognitive and behavioral disorders can arise as a result of defective synaptic plasticity, but the underlying molecular mechanisms remain incompletely understood. The work presented here suggests an important role for neuronal coronin 1 in behavioral and cognitive function via regulation of cAMP/PKA-dependent signal transduction and synaptic plasticity. We found that coronin 1 deletion or expression of an unstable coronin 1 mutant is associated with severe neurobehavioral dysfunction in both mice and humans. Mice lacking coronin 1 displayed defective socialization, enhanced grooming, defective vocalization, as well as lowered anxiety and enhanced aggression. Interestingly, the normal object recognition response together with the altered fear conditioning suggests that the defective learning and memory in the absence of coronin 1 may be a result of altered anxiety [47]. One should also note that the lower level of freezing could be a consequence of altered exploratory behavior and/or locomotor traits; However, the Y-maze analysis along with the open field and beam walking suggests that coronin 1 deficiency per se does not result in lowered exploratory activity, locomotor defects, and/or novelty seeking that are potential confounds in these behavioural tests [48]. Furthermore, coronin 1 deletion was associated with a reduction in the number of excitatory synapses as well as a virtual absence of PKA-dependent LTP at cortico-lateral amygdalary synapses, structures that are important for learning and memory [39],[49]. Together these results suggest an important function for coronin 1 in activating PKA-dependent signaling at excitatory synapses.

The specific role for coronin 1 in promoting anxiety-related synaptic plasticity via activation of cAMP/PKA signaling is illustrated by several of the findings presented here. First, the increase in ventricular size in mice lacking coronin 1 phenocopies mice in which neuronal cAMP levels are constitutively decreased [41]. Second, the membrane-permeable cAMP analogue 8-Br-cAMP was able to rescue cortico-LA long-term plasticity. Third, intra-amygdala infusion of 8-Br-cAMP resulted in a virtually complete restoration of the memory defect associated with coronin 1 deletion. Although application of 8-Br-cAMP might activate LTP in all the cells of the injection area, regardless of whether they were triggered or not during the fear conditioning, the results shown suggest that in the presence of extraneous 8-Br-cAMP the circuit specific for this fear conditioning is strengthened upon exposure to the context, thereby bypassing the cAMP/PKA defect because of coronin 1 deficiency. Furthermore, the behavioral defects are unlikely to be a consequence of developmental defects or neurodegeneration, as they occur well before the observed ventricular enlargement and neuronal loss, although more subtle morphological changes that are not detected by the methods used here cannot be ruled out. Finally, the coronin 1 neurobehavioral defects phenocopy cAMP signaling defects [41],[50]–[52]. Together these results strongly argue for a specific role for coronin 1 in the activation of cAMP signaling.

We found that the mechanism via which coronin 1 promotes cAMP production occurs via the association of coronin 1 with Gαs molecules: first, inclusion of pertussis toxin, which inactivates Gαi via ADP ribosylation, did not prevent coronin 1–dependent cAMP production. Second, coronin 1 was readily co-immunoprecipitated with Gαs but not with Gαi. Third, addition of cholera toxin, which locks Gαs proteins in their active state via ADP ribosylation, resulted in a level of cAMP production in coronin 1–deficient cells that was equivalent to the cAMP production in coronin 1–expressing cells. Therefore, neuronal coronin 1 may have evolved to specifically activate the adenylate cyclase pathway, as these enzymes are the predominant targets of Gαs proteins [53]. Possibly, exon shuffling during evolution might have endowed coronin 1 with sequence motifs that allow its binding to Gαs in order to promote adenylate cyclase activation, which, given the tissue-specific expression of coronin 1, occurs in a cell-type-specific manner.

The exact molecular mechanism by which coronin 1 modulates Gαs activity remains to be elucidated. Both coronin 1 and the Gβ subunit of the G protein heterotrimer have a seven-bladed propeller fold, sharing sequence and structural homology [33]. Also, the residues of coronin 1 that we have found to be necessary for Gαs interaction are located in a region that is homologous to the Gβ–Gαs interacting surface [54]. Thus, one possibility is that coronin 1 binds to Gαs in a similar manner as Gβ. Interestingly, several lines of evidence suggest that following receptor activation, the components of the G protein heterotrimer rearrange rather than dissociate and are maintained in a complex with the βγ subunit [55]–[57]. In this scenario, in coronin 1–expressing cells, upon stimulation coronin 1 might displace the Gβ subunit from the trimeric complex in order to modulate adenylate cyclase activity, similarly to the activation of downstream effectors by heterotrimeric G protein complexes [58]–[60]. Alternatively, coronin 1 may bind to Gαs in a manner that is distinct from Gβ, thereby promoting cAMP production (see also Figure S13). In vitro reconstitution of coronin 1–dependent adenylate cyclase activation might help to further delineate the molecular details of the role for coronin 1 in stimulating cAMP production.

Interestingly, the here described activation of cAMP/PKA signaling occurred independent of an intact F-actin cytoskeleton. These findings are consistent with earlier work using lymphoma cells, where disruption of the F-actin cytoskeleton by cytochalasin B also failed to modulate cAMP production within the time frame analyzed here [61],[62]. Although relatively little is known about the regulation of G protein–mediated adenylate cyclase activation by the F-actin cytoskeleton, these data reinforce the conclusion that, *in vivo*, coronin 1 is unlikely to mediate its function in regulating signal transduction via inducing F-actin rearrangement as shown in leukocytes [63]. However, whether or not coronin 1–mediated IP_3_/calcium signaling in naïve T cells and macrophages [23],[26],[64] occurs through the here described activation of adenylate cyclase remains to be established; although there are indications that both T cell activation as well as macrophage receptor triggering may involve G protein–coupled signaling [65],[66], cAMP mediated signaling in leukocytes is still relatively poorly understood. Also, recent work showed that upon deletion of the coronin 1 homologue in the lower eukaryote *Dictyostelium*, coronin A, the resulting developmental defect can be fully restored upon direct activation of PKA via 8-Br-cAMP incubation (Vinet et al., in press).

Given the importance of regulated cAMP signaling for appropriate neuronal functioning [13],[14],[67],[68], the here described role for coronin 1 in the activation of PKA/cAMP-dependent synaptic plasticity is fully consistent with neuronal coronin 1 being involved in the activation of cAMP production. Furthermore, the restricted expression of coronin 1 within the brain as well as to excitatory neurons may allow a subset of neuronal structures to depend for the activation of cAMP/PKA-mediated synaptic plasticity on coronin 1, while maintaining coronin 1–independent regulation of neuronal activity in other regions.

Interestingly, the coronin 1 gene is located in a genomic region on chromosome 16 in human and 7 in mouse, whose copy number variations are associated with a wide range of neuropsychiatric conditions including behavioral dysfunction and intellectual disability [16],[19],[29]. Both the mouse model as well as the patient described here exclusively lack coronin 1 expression, instead of having a deletion across a larger genomic region, which suggests a strong correlation of coronin 1 deletion and/or mutation with severe neurobehavioral abnormalities such as social deficits, stereotypic behavior, aggression, and cognitive impairment. A delay in language acquisition and behavioral as well as cognitive impairment is also described for an individual that paired a de novo deletion of 16p11.2 on the maternal allele with a 2 bp deletion in the paternal coronin 1 allele [69]. In the patient described here, the hypomorphic nature of the coronin 1 mutation may explain the differences in the neurobehavioral phenotype when compared with the mouse knock-out. With additional individuals being identified harboring coronin 1 mutations associated with cognitive deficits, it may be feasible to dissect the molecular details of the coronin 1–dependent signal transduction pathway(s) responsible for behavior and cognition.

The development of neurobehavioral and cognitive disorders is believed to result from an imbalance between inhibitory and excitatory synaptic transmission [6],[37]. Interestingly, in several models of behavioral abnormalities the E/I synapse ratio was found to be altered [70],[71] consistent with the selective expression of neuronal coronin 1 at excitatory synapses and the disturbed E/I synapse ratio in the absence of coronin 1.

In conclusion, the results presented here define coronin 1 as a regulator of behavioral processes via promoting PKA-dependent synaptic plasticity and may open novel avenues for the dissection of signal transduction pathways involved in cognitive processes.

## Materials and Methods

### Mice, Cells, Antibodies, and Reagents

Coronin 1–deficient and wild-type mice were described before [23],[26] and were used from backcross 8. All animal experimentation was approved by the veterinary office of the Canton of Basel-Stadt (approved license number 1893 and 2336) and performed according to local guidelines (Tierschutz-Verordnung, Basel-Stadt) and the Swiss animal protection law (Tierschutz-Gesetz). Hippocampal neurons were prepared as described [72] and used between day 7 and day 11. Mel JuSo cells and Mel JuSo cells transfected with coronin 1 cDNA have been described [20],[73]. H19/7 (ATCC: CRL-2526) and N1E-115 (ATCC-CRL-2263) cells were from ATCC. H19/7 cells were cultured in DMEM supplemented with 10% fetal bovine serum, 0.2 mg/ml G418, and 0.001 mg/ml puromycin in a 34°C incubator with 5% CO_2_. N1E-115 cells were cultured in DMEM supplemented with 10% fetal bovine serum at 37°C and 5% CO_2_. Isoproterenol and cholera toxin were from Sigma and roscovitine from Cayman or Sigma chemicals. Antibodies used were from the following sources: rabbit anti-coronin 1 has been described before [20],[32], mouse anti-coronin 1 was from Abnova, mouse anti-neurofilament medium chain was from Sigma, mouse anti-GAD67 was from Chemicon, mouse anti–synapsin 1 was from BD Sciences, anti-tubulin (E7) was developed by M. Klymkowsky and obtained from the Developmental Studies Hybridoma Bank at the University of Iowa, anti-cAMP from Santa-Cruz Biotechnologies, anti-PKA substrate antibody from Cell Signaling, anti-P-CREB antibodies were from Abcam, and mouse anti-VGAT and mouse anti-Vglut1 antibodies were from Synaptic Systems. Mouse anti-histone antibody was from Santa Cruz Biotechnology and DRAQ5 was from Biostatus. Forskolin was from Tocris, and Rolipram was from Sigma. Neurotrace red and Dapi (brain stain kit) were from Life Technologies. Rolipram was from Sigma. The ICUE3 plasmids were kindly obtained from Dr. Jing Zhang (Johns Hopkins University). For cAMP ELISA, cells were seeded at a density of 2×10^6^ cells per well on a six-well plate and allowed to adhere overnight and in the case of Mel JuSo cells transfected with WT Cor 1 HA pCB6 or control plasmid (pMax GFP or pCB6) and used after 48 h. For live cell imaging, coronin 1–expressing or wild-type Mel JuSo cells [20] were seeded at a density of 20,000 cells per well on an eight-well chambered coverslip (0.11 mm) and cultured overnight. For mutagenesis and cell transfection, see Text S1 “Materials and Methods.”

### Behavioral Analysis

Data shown in Figure S1C,F,G, were performed according to established and standardized protocols at the Institut Clinique de la Souris, Strasbourg, France. Twelve wild-type and 12 coronin 1–deficient male mice aged 15–20 wk were used for this study. Mice were allowed to acclimatize for 2 wk prior to analysis using the following tests: For Y-maze spontaneous alternation, each mouse (males, 17–18 wk) was placed at the end of one arm of the Y-maze, the head directed to the walls, and allowed to explore freely the apparatus for 5 min, with the experimenter out of the animal's sight. Alternations are operationally defined as successive entries into each of the three arms as on overlapping triplet sets. Percent spontaneous alternation performance was defined as the ratio of actual (total alternations) to possible alternations (total arm entries, 2)×100. Total arm entries and the latency to exit the starting arm were also scored as an index of ambulatory activity and emotionality in the Y-maze, respectively (*n* = 12 per group).

For dark versus light preference, a rectangular, black Plexiglas box of dimension 80 cm×30 cm divided into two compartments with one compartment well lit and the other kept dark [5],[74] was used (see Text S1 “Materials and Methods” for analysis). For grooming analysis, a rectangular, black Plexiglas box of dimension 80 cm×30 cm was used that was divided into two equal compartments using a transparent Plexiglas partition placed in the middle [27],[28]. Both the compartments were covered with a red Plexiglas lid to avoid any external interference. For analysis, see Text S1 “Materials and Methods.” Resident-intruder test was performed as described [75],[76] and further detailed in Text S1 “Materials and Methods.” For analysis of social dominance using the tube displacement test, mice (males, 14–26 wk) were tested using a plastic tube [30 cm long, 3.5 cm Ø and cleft at the top (1.5 cm)] with their openings closed with a carton plug and connected to side chambers [10 cm×6 cm×10 cm (LxBxH)] [76]–[78]. Analysis was carried out as described in Text S1 “Materials and Methods.” Regarding the elevated plus maze test, this is a conflict test based on a natural tendency of mice to actively explore a new environment, versus the aversive properties of an elevated open runway [27],[79] (see Text S1 “Materials and Methods”). For the olfactory habituation-dishabituation analysis to assess olfactory function [5], mice (wild-type or coronin 1–deficient, males, 13–20 wk) were shifted and acclimatized to the test room at least 30 min prior to the analysis (see Text S1 “Materials and Methods”). The three chamber social interaction assay conducted as described [79] using an apparatus consisting of a polycarbonate box with removable partitions separating the box into three chambers (40.5×60.0×22.0 cm; Noldus Information Technology, the Netherlands). The partitions had openings that allowed the animal to move freely from one chamber to another. Both the side chambers contained one stranger cage (steel wire mesh cage, 11 cm diameter, individual wire mesh separated by 1.1.cm, height 20 cm) with a Plexi glass lid. For details on the analysis, see Text S1 “Materials and Methods.” Vocalization was analyzed using a rectangular, black Plexiglas box of dimension 80 cm×30 cm divided into two equal compartments using a transparent perforated Plexiglas partition placed in the middle (multiple perforations, diameter 1 cm) (see Text S1 “Materials and Methods”). For fear conditioning, acquisition and retrieval of cued fear conditioning took place in two different contexts (context A and B), and analysis was performed as described in Text S1 “Materials and Methods.”

### Cell and Tissue Lysis and Immunoblotting

Microdissected tissue from wild-type and coronin 1–deficient mice (age 6–8 wk) were lysed in Triton-X 100 buffer (20 mM HEPES pH7.4, 100 mM NaCl, 5 mM MgCl_2_, 1% Triton X-100) with 0.2% SDS containing protease and phosphatase inhibitors (Roche) at 4°C. After protein determination (BCA, Pierce), equal amounts of protein (15 µg per lane) were separated by 12.5% SDS-PAGE, transferred onto nitrocellulose, and probed using the mentioned antibody followed by HRP-labeled secondary antibodies and developed using an enhanced chemi-luminescence imager (Fuji) as described before [73].

### Immunofluorescence Analysis

In brief, hippocampal neurons (7 d cultures) were washed with PBS followed by fixation with PFA (4% in Dulbecco's PBS) immediately at room temperature for 20 min, followed by washing and saponin permeabilization (15 min 0.5%) at room temperature. After 30 min blocking with 2% BSA in PBS, the cells were incubated with primary antibodies (1∶1,000, 1 h RT) of rabbit anti–coronin 1 and mouse anti–synapsin 1 or mouse anti–coronin 1 followed by Alexa Fluor 488 or −568 labeled secondary antibody incubation at room temperature for 1 h, respectively. Preparations were analyzed on a Zeiss LSM 510 Meta Confocal Laser Scanning microscope.

### Immunohistochemistry of Brain Sections

Wild-type and coronin 1–deficient mice (age 6–8 wk) were anesthetized in a 4% isoflurane chamber, decapitated, and the brains dissected out immediately and transferred stepwise into 2-methylbutane solution supercooled to −40°C. Once frozen, the sections were transferred to −80°C until sectioning. Prior to sectioning, the brains were warmed to −20°C for 30 min and mounted on a cryostat (Microm HM 560 or Leica CM1950). Thin brain sections (12 micron) were transferred onto superfrost glass slides (Thermo Scientific), air dried, fixed with 4% paraformaldehyde for 10 min at room temperature followed by a cycle of dehydration with 70%, 95%, and 100% ethanol and rehydration in the reverse order. Alternatively, brain sections from C57BL/6 or CD1 mice (Zyagen) were used for staining. The samples were then subjected to antigen retrieval in 10 mM citrate phosphate buffer (10 mM Citric acid pH6, 0.05% Tween 20) overnight at 60°C followed by blocking with 5% FBS for 2 h. For incubation with primary antibodies, rabbit anti–coronin 1 primary antibody (serum 1002, 1∶1,000 dilution) or rat anti–coronin 1 serum (1∶100) along with mouse anti-neurofilament medium chain or chicken anti-neurofilament heavy chain antibody, rabbit anti-pCREB, and mouse anti-histone antibody in PBS with 2% fetal bovine serum were layered over the samples for 4 to 16 h. After three washes with PBS containing 2% fetal bovine serum, anti-rabbit Alexa Fluor 488 (1∶200) or anti-rat Alexa Fluor 488 (1∶200) and anti-mouse-Alexa Fluor 546 or anti-chicken Alexa Fluor 563 tagged secondary antibodies (1∶200) were added. One hour postincubation, the slides were washed three times and the slides sealed with a coverslip in the presence of antifade (BioRad) and viewed under a confocal microscope (Carl Zeiss LSM 510Meta or LSM 700). Quantitation was carried out from captured images of brain sections and analyzed using ImageJ (NIH) software.

### Serial Block Face Scanning Electron Microscopy

Hippocampal tissue was processed according to Knott et al. (2002) [80]. In brief, wild-type or coronin 1–deficient mice (*n* = 3) were transcardially perfused with 2% paraformaldehyde and 2% glutaraldehyde. Then the brain was removed and cut with a vibratome (Leica). Sections containing the CA1 region of hippocampus were chosen, stained in 1% osmium, and 1% uranyl acetate before being dehydrated in a successively increased concentration of ethanol. Sections were then flat-embedded in the Epon (Fluka) resin between two glass slides. The material was subsequently processed for ultrastructural analysis using the 3view microtome (GATAN, UK) inserted in a QUANTA 200 VP-FEG scanning electron microscope (FEI, Netherlands) as described [81]. In brief, volumes of 500 cubic microns located in the CA1 area for each animal were imaged, and synapses were classified as excitatory or inhibitory according to established criteria [80] and based on their asymmetric (excitatory) or symmetric (inhibitory) aspects.

### Electrophysiology

#### Amygdala

Standard procedures were used to prepare 330-µm-thick coronal slices from 3- to 4-wk-old male wild-type or coronin 1–deficient mice (C57BL/6J background) following a protocol approved by the European and French guidelines on animal experimentation. Briefly, the brain was dissected in ice-cold artificial cerebrospinal fluid (ACSF), mounted on an agar block, and sliced with a vibratome (Leica VT1200s; Germany) at 4°C. Slices were maintained for 45 min at 35°C in an interface chamber containing ACSF equilibrated with 95% O2/5% CO_2_ and containing (in mM) 124 NaCl, 2.7 KCl, 2 CaCl_2_, 1.3 MgCl_2_, 26 NaHCO_3_, 0.4 NaH_2_PO_4_, 18 glucose, 4 ascorbate, and then for at least 45 min at room temperature before being transferred to a superfusing recording chamber. Whole-cell recordings from LA principal neurons were performed at 30–32°C in a superfusing chamber as previously described [82]. Neurons were visually identified with infrared video microscopy using an upright microscope equipped with a 60× objective. Patch electrodes (3–5 MΩ) were pulled from borosilicate glass tubing and filled with a low-chloride solution containing (in mM) 140 Cs-methylsulfonate, 5 QX314-Cl, 10 HEPES, 10 phosphocreatine, 4 Mg-ATP, and 0.3 Na-GTP (pH adjusted to 7.25 with CsOH, 295 mOsm). For current-clamp experiments, Cs-methylsulfonate was replaced with equimolar K-gluconate. All experiments were performed in the presence of picrotoxin (100 µM). Monosynaptic EPSCs exhibiting constant 10%–90% rise times and latencies were elicited by stimulation of afferent fibers with a bipolar twisted platinum/10% iridium wire (25 µm diameter). Data were recorded with a Multiclamp700B (Molecular Devices, USA), filtered at 2 kHz, and digitized at 10 kHz. In all experiments, series resistance was monitored throughout the experiment, and if it changed by more than 15%, the data were not included in the analysis. Thalamo-LA LTP was induced by pairing 15 bursts of three postsynaptic action potentials with three presynaptic stimulations at 30 Hz. Pre- and postsynaptic stimulations were separated by 10 ms [38]. Cortico-LA LTP was induced by pairing postsynaptic depolarization (8 s, +30 mV) with presynaptic stimulation (80 stimuli at 10 Hz) [83]. Data were acquired and analyzed with pClamp10.2 (Molecular Devices). Changes were quantified by normalizing and averaging EPSC amplitudes or EPSP slopes during the last 10 min of the experiments relative to the 5 min of baseline prior to LTP induction or drug application. All values are given as means ± standard error of the mean (SEM). Mean values were compared between genotypes using either unpaired Student's *t* test or Mann–Whitney (MW) test as appropriate. Preincubation of slices with 8-Br-cAMP (Sigma; 0.5 mM for 2 h before recordings) was performed in immersion chambers. Concentration values were taken from previous studies [84],[85].

#### Hippocampus

Hippocampal slices were obtained from wild-type and coronin 1-deficient mice. Animals were sacrificed by cervical dislocation, and the brain was then quickly removed and placed in ice-cold artificial CSF (aCSF) bubbled with carbogen (95% CO_2_/5% O_2_) and in which NaCl was replaced with sucrose (in mM: 220 sucrose, 1,99 KCl, 26 NaHCO_3_, 1,5 NaH_2_PO_4_, 10 glucose, 0,2 CaCl_2_, and 6 MgCl_2_ pH 7.4, osmolarity ∼320 mOsm). Transverse 350-µm-thick slices were cut using a vibratome (Leica 1200S) and transferred to a heated (33°C) holding chamber containing oxygenated (95% CO_2_/5% O_2_) standard aCSF (in mM: 124 NaCl, 3 KCl, 26 NaHCO_3_, 1.25 NaH_2_PO_4_, 10 glucose, 2 CaCl_2_, and 1 MgCl_2_, osmolarity ∼300 mOsm) for 30 min, and then subsequently maintained at room temperature for recordings. Recordings were performed as for amygdala slices in the presence of 2.5 µM of tetrodotoxin citrate (Ascent Scientific). Analysis of synaptic currents was done using a template routine under pClamp10.2 (Molecular Devices).

### Primary Hippocampal Culture cAMP Assay

Wild-type or coronin 1–deficient pups (P0 to P2) were sacrificed and their brain hippocampal regions microdissected and rapidly processed further by trypsinization and tituration for single cell preparation. The cells were initially plated in plating medium (DMEM supplemented with fetal bovine serum 10%, glutamax 1%, penicillin 100 units/ml, streptomycin 100 µg/ml, and glucose 1.25%) for 2–6 h and subsequently in maintenance medium (Neurobasal supplemented with B27 2%, glutamax 1%, penicillin 100 units/ml, and streptomycin 100 µg/ml). The cultures were treated with cytarabine (10 µM, Sigma) from day 4 of the culture and used between day 7 to 14 for cAMP determination. For cAMP analysis, the cells were cultured in plain neurobasal medium for 2 h in the presence of the cAMP-specific phosphodiesterase inhibitor rolipram (100 µM, Sigma). Subsequently, cells were either left unstimulated or stimulated as indicated in the presence of rolipram and lysed with the provided cAMP ELISA lysis buffer (cAMP parameter kit, R&D Systems). The lysate was centrifuged at 9400×*g* for 10 min/4°C, and the supernatant assayed for total protein amounts by BCA assay. Subsequently equal protein amounts of the lysate were analyzed for cAMP levels as per the manufacturer's protocol (cAMP parameter kit, R&D Systems).

### Brain cAMP Assay

Wild-type or coronin 1–deficient mice (4–5 wk old) were sacrificed and their brains were immediately dissected out and rapidly processed further at 4°C. The amygdalary regions were microdissected, homogenized, and lysed with the provided cAMP ELISA lysis buffer. The lysate was centrifuged at 9400×*g* for 10 minutes/4°C and the supernatant assayed for total protein amounts by BCA assay. Subsequently equal protein amounts of the lysate were analyzed for cAMP levels as per the manufacturer's protocol (cAMP Parameter Kit, R&D Systems).

### PKA Assay

PKA activity was measured from 6–8-wk-old wild-type or coronin 1–deficient mice using the Peptag nonradioactive cAMP-dependent protein kinase assay (V5340; Promega, three animals per group). Manually dissected cerebral structures were snap-frozen in liquid nitrogen. All samples were treated together, in duplicates, and corrected for protein concentration (per milligram of total protein). Samples were loaded in the same electrophoretic gels. Protein density was controlled under UV light and simultaneously measured using the Genetools 1.0 software (SYNGENE, Frederick, MD). The basal PKA activity represents the difference between the ratios of phospho-/nonphospho forms. A series of experiments was done in the presence of 1 µM cAMP to estimate the maximal PKA activity.

### Magnetic Resonance Imaging (MRI)

All MRI was performed with a 7-Tesla Bruker MRI imaging spectrometer (Bruker Biospec 70/21) using a mouse brain surface coil (Rapid Biomedicine GmbH, Germany). For in vivo MRI scanning, mice were anesthetized with 1.5%–2% isoflurane and secured using a head holder to reduce motion artifacts. We obtained 12 (Figures 4F,G and S7B) or 18 (Figure S7A) coronal T2-weighed images through the entire mice brains using a 2-dimensional multislice spin echo sequence with the following parameters: for the data shown in Figures 4F,G and S7B, 12 coronal T2-weighed images were obtained (Paravision software PV 3.0.2) using a 2-dimensional multislice spin echo sequence with the following parameters: field of view, 20 mm, acquisition matrix, 256×256; slice thickness, 0.6 mm; time of repetition, 3130.9 ms; Echo, 1; TE effective 1, 80 ms; number of averages, 8. The ventricular areas of all coronal MRI images from each mouse were quantified using the ImageJ program and converted to total pixels per mouse. The data shown are the average ventricular area represented in pixels with SEM (*n* = 6 per group). For the data in Figure S7A, 18 coronal T2-weighed images were obtained using Paravision software PV 4.0, with the following deviations: acquisition matrix, 512×280; time of repetition, 4,691.8 ms; TE effective 1, 76.6 ms, with the other parameters being as described above. The ventricular areas of the coronal MRI images were quantified using ImageJ and converted to total pixels per mouse. Data shown represent the average ventricular area in pixels with SEM (*n* = 4–6 mice per genotype).

### Cell Stimulation and cAMP ELISA

Cells were subjected to 2 h of serum starvation and either left unstimulated or stimulated with isoproterenol (5 or 10 µM for 4 min or as stated) in the presence of rolipram (100 µM, where indicated) followed by a wash in ice-cold PBS and processed for cAMP analysis (R&D Systems) according to the manufacturer's protocol. For the cholera toxin stimulation experiments, coronin 1–expressing Mel JuSo cells or control cells were seeded on a six-well plate and allowed to grow to ∼80% confluence, starved in Opti-MEM for 1 h (with 100 µM Rolipram), followed by stimulation with cholera toxin (1 µg/mL) to the starving cells and further incubation for another 1 h at 37°C. Cells were left untreated or stimulated with isoproterenol (10 µM) for 4 min before cAMP production was measured. Values were normalized to total protein amounts (BCA analysis) and converted to pmol cAMP/mg protein. Data shown are representative of three independent experiments.

### Live Cell Imaging

Cells were transfected with the ICUE3 plasmid (from Dr. Jing Zhang, Johns Hopkins University) using Fugene 6 following the manufacturer's protocol. After 24 h or 48 h of transfection, the cell culture medium was changed to phenol red-free opti-MEM (Invitrogen) and live cell imaging was performed using a Zeiss Cellobserver (63×, Oil). FRET measurement and analysis was carried out as described below.

### FRET Measurement and Analysis

Cells were excited using a band pass filter (445/25 nm, for CFP), and the emission signals from both the CFP (band pass filter 480/22 nm) and the YFP (long pass filter 530 nm) channel were collected by two different EMCCD cameras (Evolve) simultaneously. Isoproterenol (5 µM) was added at the time point of 25 s. After acquisition, the background of both CFP and YFP channels was corrected, and the images of both CFP and YFP channels were subjected to ratiometric analysis. The ratiometric imaging of the CFP/YFP channels was calculated pixel by pixel. The ratiometric time course was calculated by the average fluorescence intensity of the entire cell in both CFP and YFP channels.

### Immunoprecipitation

Following cellular stimulation, cells were homogenized in buffer A [20 mM HEPES-NaOH pH 7.8, 30 mM NaCl, 0.5 mM DTT, 0.2 mM PMSF, 1 mM EDTA, protease and phosphatase inhibitor cocktail (Thermo Scientific)], cooled on ice for 10 min, followed by centrifugation (5 min at 1,700×*g*). The pellet was resuspended in buffer A containing 1% sodium-beta-D-maltoside, incubated on ice for 20 min, and centrifuged at 14,000×*g* for 5 min and pooled with the first supernatant. Immunoprecipitation was carried out using antibodies coupled to Dynabeads Protein G (Invitrogen) using DMP crosslinking (Abcam) according to the manufacturer's protocol. Immunoprecipitated samples were washed four times with buffer A followed by elution in Laemmli sample buffer and analyzed by SDS PAGE (10% or 12%) and immunoblotting.

### Analysis of β2-Adrenergic Receptor Expression

To analyze surface expression of β2-adrenergic receptor levels in coronin 1–expressing and nonexpressing cells, Mel JuSo cells (lacking coronin 1) were labeled with Celltrace Violet (Invitrogen) following the manufacturer's protocol and mixed with an equal number of coronin 1–expressing Mel JuSo cells [20] and fixed with 4% paraformaldehyde for 20 min at room temperature. Following this, the cells were washed, blocked with 2% Fetal Bovine Serum containing PBS for 30 min, and incubated with goat polyclonal β2-adrenergic receptor antibody (1∶500, Abcam) for 1 h. Following washes, the cells were incubated with donkey anti-goat Alexa fluor 488 antibody (1∶1,000, Invitrogen) for 45 min and washed again before being taken up for FACS analysis using BD FACS Canto II with UV laser line 405 nm (for celltrace violet) and laser line 488 (for β2-adrenergic receptor). The profiles were analyzed and processed using Flowjo software. The data shown are representative of three independent experiments.

### RT-PCR

RT-PCR analysis for components of the GPCR/cAMP pathway was performed in Mel JuSo cells, as the coronin 1–dependent cAMP production in Mel JuSo cells equals that in neurons or macrophages and cultured cell systems are less prone to variation compared to mouse organ preparations. Total RNA was isolated from 5×10^6^ cells, expressing or lacking coronin 1 (Mel JuSo), using a Qiagen RNA isolation kit in accordance with the manufacturer's protocol. RNA integrity was analyzed employing the RNA 6000 Nano Assay Kit (Agilent Technologies). Reverse transcription (RT) reactions were set up according to the manufacturer's protocol with 0.65 µg total RNA, Super RT enzyme (HT Biotechnology), and oligo-dT primers (Invitrogen). Polymerase chain reactions using various specific primers (Table S4) and cDNA templates from the RT reactions consisted of 40 cycles of 96°C for 30 s, 56°C for 30 s, and 72°C for 60 s using a RT-PCR Power SYBR Green cocktail (Applied Biosystems) using Rotor gene 6000A (Corbett Research). As a control, PCR reactions were performed concurrently with primers for human transferrin receptor along with appropriate RT minus controls. PCR efficiency was determined by performing an eight fold dilution series of four steps in duplicates (100% for TFRC). Threshold cycles (crossing point) were determined using Rotor-Gene software version 6.1. Expression levels were normalized to TFRC [86]. Fold differences were calculated using the delta-delta Ct method [87]. Statistical significance was calculated using Student's *t* test. Three independent experiments were performed. Results shown are from quadruplicate biological samples per cell type and quadruplicate RT-PCR runs per sample (4×4 = 16 runs per gene) normalized against TFRC gene. Note that for ADCY2, ADCY4, ADCY5, ADCY8, ADCY9, ADRB1 and ADRB3 no signal was obtained, suggesting lack of expression.

### Protein Purification

Coronin 1 purification was performed from macrophages essentially as described before [32]; prior to purification, cells were treated with 5 µM Roscovitine for 24 h. Ten confluent 15 cm tissue culture plates of macrophages were harvested and washed using ice-cold PBS 3 times and lysed in 0.5% Triton X-100 buffer (50 mM Tris-HCl, 137 mM NaCl, 10 mM EGTA, 0.5% Triton X-100), containing protease inhibitor (Roche, Mini complete protease inhibitor cocktail tablets and 1 mM PMSF added briefly before use) on ice for 30 min. Afterwards, the nucleus and unlysed cells were sedimented (14,000×*g*, 15 min at 4°C) and the supernatant used for coronin 1 purification. After the preparation of the column, the cell lysate was loaded twice on the anti–coronin 1 affinity column [32], washed using 15 ml washing buffer (50 mM Tris-HCl, 150 mM NaCl, pH 8), and eluted by 5 ml elution buffer (0.1 M glycine, pH 2.5). Then, the eluate containing purified coronin 1 was immediately neutralized by 1 M Tris pH 10. Following purification, the buffer was changed to kinase assay buffer (60 mM HEPES-NaOH, 3 mM MgCl_2_, 3 mM MnCl_2_, 3 µm Na-orthovanadate, 1.2 mM DTT, 50 µg/ml PEG 20,000, pH 7.5) and the eluate concentrated to 0.5 mL using centrifugal filter units (Millipore, Amicon Ultra Centrifugal Filters) according to the manufacturer's protocol. The purity of the coronin 1 was analyzed by SDS-PAGE (10%) followed by Coomassie staining.

For the surface plasmon resonance experiments (see below), coronin 1 or the coronin 1 mutant in which Arg-69, Lys-20, Lys-355, and Glu-102 had been mutated to alanine were expressed as HA-tagged molecules in Mel JuSo cells using the lipofectamine LTX (Invitrogen/Life Technologies) protocol according to the manufacturer's instructions. Cells were harvested after 48 h and washed with ice-cold PBS 3 times followed by lysis on ice in cell lysis buffer (50 mM HEPES-NaOH pH 7.5, 150 mM NaCl, 1% Triton-X 100, 0.5% NaDOC, 2 mM MgCl_2_, 1 mM CaCl_2_, 0.5 mM EDTA, 0.5 mM PMSF, 1 tablet of Roche complete EDTA free protease inhibitor cocktail) for 30 min. The lysate was centrifuged at 21,000×*g* at 4°C for 20 min. Supernatants were collected, filtered (0.2 µm), and subjected to protein purification. For purification, an anti-HA bead column (Pierce) was prepared and washed with 10 ml 100 mM Tris-HCl pH 7.5, followed by 10 ml of buffer A (50 mM HEPES-NaOH pH 7.5, 150 mM NaCl, 2 mM MgCl_2_, 1 mM CaCl_2_, 0.5 mM EDTA) followed by 10 ml cell lysis buffer. Afterwards, the cell lysate was loaded on the anti-HA column and the column was sealed and rotated at 4°C for 2 h. Subsequently, the lysate was collected and reloaded on the anti-HA column for another 3 times. After the lysate loading, the column was washed with 10 ml cell lysis buffer followed by 10 ml of buffer A. The HA-tagged coronin 1 proteins were eluted with 2 ml HA peptide (0.5 mg/ml, Pierce) in a buffer containing 150 mM NaCl, 2 mM MgCl_2_, 1 mM CaCl_2_, 50 mM HEPES-NaOH pH 7.5. Fractions were collected and analyzed by silver staining as well as Western blotting as described above.

### Gαs Purification

For Gαs purification, the Gαs proteins, both short and long form, were expressed using in-house constructed expression vectors [88] from the T7 promoter. The N terminus was fused to a 6×His-tagged *E. coli* thioredoxin A. In addition, a short form construct with a C-terminal 6×His tag was prepared. The constructs were verified by DNA sequencing (GATC, Germany). The proteins were produced in *E. coli* NiCo21 (DE3) cells (New England Biolabs) in ZYM-5052 auto-induction medium [89] overnight at 20°C. Subsequently, they were purified by Ni-NTA-chromatography and gel filtration in 100 mM Tris-Cl pH 8.0. Total yields were about 2 mg pure protein from 6l ZYM5052 culture.

### Surface Plasmon Resonance

Purified Gαs was immobilized on a Series S Sensor Chip NTA (GE Healthcare, BR-1005-32) as follows: The Series S Sensor Chip NTA sensor chip was precleaned by washing with 0.35 M EDTA in water followed by activation of the chip with 0.5 mM NiCl_2_. The mixture (1∶1) of 1-ethyl-3-[3-dimethylaminopropyl]-carbodiimide hydrochloride (EDC, 0.4 M) and N-hydroxysuccinimide (NHS, 0.1 M) solution was injected into the NTA chip (480 s, flow rate 5 µl/min) to initiate the reaction. Afterwards, the purified Gαs (10 µg diluted in running buffer, 150 mM NaCl, 2 mM MgCl_2_, 1 mM CaCl_2_, 50 mM HEPES-NaOH pH 7.5, 1 mg/ml BSA, 0.05% NP40) was injected into the chip. Finally, the reaction was quenched by injecting ethanolamine solution (1 M in water) for 7 min (flow rate 5 ml/min). The unbound protein was further removed by washing with 0.35 M EDTA in water for 90 s. The chip was equilibrated by using the running buffer for 40 min before use. Purified coronin 1 protein or coronin 1 mutant protein was diluted in running buffer at the concentrations indicated and injected into the Gαs immobilized SPR chip with the following parameters: contacting time, 180 s; dissociation time, 360 s; flow rate, 30 ml/min. A regeneration step was included after each cycle by injecting for 60 s a regeneration buffer (1.5 M NaCl in running buffer), followed by a 360 s stabilization step in running buffer. The binding level and binding kinetics were collected and analyzed by the software provided by the supplier.

### Cannula Implantation and 8-Br-cAMP Infusion

Under continuous anesthesia with isoflurane, mice were positioned in a stereotaxic apparatus (David Kopf Instruments, Tujunga, CA). Stainless steel guide cannula (26 gauge; Plastics-One, Roanoke, VA) were bilaterally implanted above amygdala (from Bregma position, anteroposterior [AP] −1.6 mm, mediolateral [ML] ±3.3 mm, dorsoventral [DV] −3.2 mm). Cannulas were anchored to the skull with dental cement (Super-Bond, Sun Medical Co. Lt). In the end, the mice waked up on a 35°C warm pad, and dummy cannula were inserted into the guide to reduce the risk of infection. To keep mice not stressed during the injection, the mice were trained with a dummy cannula remove and insertion protocol, and they could move freely in their cage. An infusion cannula (33 gauge; connected to a 1 µL Hamilton syringe via polyethylene tubing) projected out of the guide with 1 mm to target the amygdala. The 8-Br-cAMP sodium salt (Sigma; diluted in saline with 1.5 µg in 300 nL) was infused bilaterally at a rate of 0.1 µL per min in a volume of 300∼350 nL per side [45],[90], which was controlled by an automatic pump (Legato 100, Kd Scientific Inc., Hilliston, MA). The single injection group was injected 30∼45 min before acquisition, and the second injection group was performed both 30∼45 min before and 2.5∼3 h after acquisition. To allow penetration of drug, the injector was maintained for an additional 3 min. Then the mice were transferred back to the cages for additional rest. In the end, to analyze the location and extent of the injection area, brains were infused with a fluorophore BODIPY TMR-X (Invitrogen; 5 mM in PBS 0.1 M, DMSO 40%). Then slices (60 µm) were imaged using a 5× epifluorescence microscope (Leica DM5000). Mice were preserved in the analysis only if one side of bilateral injections was precisely targeted or if both saturated areas covered more than 25% of the amygdala.

### Statistical Analysis

Statistical analysis was performed using GraphPad Prism (version 4.0) and SPSS (version 20). Significance was considered at *p*<0.05 if not otherwise specified. *p<0.05, **p<0.01, ***p<0.001.

## Supporting Information

Figure S1 **Behavioral phenotyping of wild-type and coronin 1–deficient mice.** (A) Light versus dark preference. Number of transitions between the compartments (*n* = 14 WT, 16 cor 1 −/− mice). *P*<0.05, Student's *t* test. Latency to enter bright compartment (*n* = 14 WT, 16 cor 1 −/− mice). *P* = 0.091, Mann–Whitney U test. (B) Elevated plus maze, number of arm entries. No significant difference between wild-type and cor 1 −/− mice (*n* = 16 WT, 15 cor 1 −/− mice). (C) Locomotor activity. Motor function was tested in wild-type and cor 1 −/− animals using a Y-maze (*n* = 12 WT, 12 cor 1 −/− mice). (D) Mean CS+-induced freezing during acquisition of cued fear conditioning in wild-type (WT; *n* = 12) and cor 1 −/− (*n* = 11) mice. (E) US-sensitivity. Foot-shock–induced flinching behavior and vocalization were not different between coronin 1 −/− (*n* = 6) and wild-type (*n* = 5) mice (*p*>0.05, Student's *t* test for both behaviors). (F, G) Open field and novel object recognition (*n* = 12 WT, 12 cor 1 −/− mice). *p<0.05, Student's *t* test. **p<0.01, Student's *t* test. See Table S1 for additional statistics.
(TIF)

Figure S2 **Social interaction of wild-type and coronin 1–deficient mice.** (A) Similar olfactory function of coronin 1 −/− mice and wild-type mice as analyzed using various cues (soc, social cue) (ns; RM-ANOVA). *n* = 10 WT and 10 cor 1 −/−. (B–E) Three-chamber socialization assay. (B) Number of entries in the chambers indicated; E, empty; L, left; R, right; S1 and S2, stranger mouse 1 and 2; ses I, session I (E versus S1); ses II, session II (S2 versus S1). (C–E) Time spent in chamber for the cages in habituation session (C), session I (D), and session II (E). Coronin 1 −/− mice show a reduced sociability in session 1 and a reduced social novelty in session 2 relative to wild-type mice. RMANOVA, **p<0.01 and ***p<0.001. *n* = 16 WT and 16 Cor 1 −/−. (F) Reduced social interaction of cor 1 −/− mice as assessed by modified Paylors partition method. Student's *t* test, *p*<0.0001. *n* = 13 WT, 14 cor 1 −/− (for Singletons) 13 WT and 16 Cor 1−/− (for stranger). See Table S1 for additional statistics.
(TIF)

Figure S3 **A point mutation in human coronin 1 causes severe cognitive disabilities.** (A) Alignment of yeast and zebrafish coronin and murine and human coronin 1. (B) Location of the valine-methionine mutation at position 134. (C) Cell lysates from HEK293 cells transfected with cDNA encoding either wild-type coronin 1, coronin 1^V134M^ or left untransfected (lane 3), were analyzed by SDS-PAGE and immunoblotting using anti–coronin 1 antiserum (upper panels) or anti-actin antibodies (lower panels). Left, untreated. Right, proteasome-inhibitor-treated. (D) Motor skills and language acquisition. (E) Neurological characterization of the patient. (F) Scores on the K-ABC for the patient at 8 y and 8 mo of age compared to normal controls.
(TIF)

Figure S4 **Localization of coronin 1 by immunofluorescence analysis in wild-type and coronin 1–deficient sections and cultured neurons.** (A–C) Characterization of coronin 1 expression in wild-type and coronin 1–deficient hippocampi (A), dorsal root ganglion (B), and primary hippocampal cultures (C) using coronin 1 rabbit anti-serum followed by Alexa Fluor 488 (A), Alexa Fluor-568 (B), or Alexa Fluor 647 (C) as well as the other markers indicated. Scale bar, 30 µm (A), 50 µm (B), and 20 µm (C).
(TIF)

Figure S5 **Altered mEPSC frequencies in the absence of coronin 1.** (A) E/I ratio [*n* = 13 cells for wild-type (grey) and *n* = 12 cells for coronin 1–deficient (red)]. *p*<0.01, Mann–Whitney U test. (B) Excitatory and inhibitory transmission in the hippocampus CA1 pyramidal neurons. Scale bars, 20 pA and 0.5 s (mIPSC) and 5 sec (mEPSC). (C) Miniature EPSC and IPSC's amplitudes and decay times recorded from wild-type and coronin 1–deficient hippocampus; *n* = 13 wild-type, 12 Cor1 −/−, *p*>0.05 (mEPSC), *p*>0.05 (mIPSC), Student's *t* test, see Table S1.
(TIF)

Figure S6 **Electrophysiology of thalamo- and cortico-LA afferents in wild-type and coronin 1–deficient mice.** (A) Minimal stimulation of afferent axons typically results in well-separated EPSC responses (successes) and failures. Scale bars, 30 pA and 5 ms. (B) Consistent with the double stimulation of a single axon, the release probability (success rate) is increased when a second stimulation occurs at a short interval (50 ms). Scale bars, 30 pA and 15 ms. (C, D) Summary graphs illustrating normal amplitude of thalamo- and cortico-LA minimal responses in coronin 1–deficient mice (*n* numbers are indicated on bar graphs; n.s., not significant), *p*>0.05, Student's *t* test. (E) The cortico-LA AMPA receptor-mediated component of synaptic transmission was quantified at −70 mV (peak amplitude). The NMDA receptor-mediated component of synaptic transmission was quantified at +50 mV (amplitude at 100 ms after stimulation). Scale bars, 50 pA and 50 ms. (G) Cortico-LA NMDA/AMPA ratios did not differ between wild-type (WT) and coronin 1–deficient (Cor1 −/−) mice. Scale bars, 50 pA and 50 ms. *n*, numbers are indicated on bar graphs, *p*>0.05, Student's *t* test. (F, H) Same as (E, G) for thalamo-LA synapses, *p*>0.05, Student's *t* test.
(TIF)

Figure S7 **MRI analysis of brain ventricles and histology in wild-type and coronin 1–deficient animals.** (A) Ventricle sizes in the presence and absence of coronin 1 as analyzed by MRI of mice aged either less than 6 wk or ∼32 wk. *n* = 4. Shown are mean ventricular size (left, right, and middle) ± SEM. *p*<0.05, 0.01, and 0.05 for left, central, and right ventricle, respectively (Student's *t* test). (B) Sequential MRI imaging of a representative wild-type and coronin 1–deficient mouse. (C, Left panels) CA1 hippocampal regions of age-matched male wild-type and coronin 1–deficient mice stained with neurotrace red and Dapi and imaged using a confocal microscope (Zeiss LSM 700). Scale bar,20 µm. (Right panels) Quantitation of the neurotrace red-positive cell numbers in the CA1 hippocampal region (*n* = 3 mice per genotype). The data (mean ± SEM) are represented as the numbers of neurons per 100-µm linear length of medial CA1 (*n* = 10–12 regions from three different mice in each group). *p*<0.01 (Student's *t* test), see also Table S1.
(TIF)

Figure S8 **Effect of coronin 1 on expression of components of the beta-adrenergic receptor signaling pathway.** (A) FACS analysis of surface expression of β2-adrenergic receptor in wild-type and coronin 1–expressing Mel JuSo cells (upper panel) and bone-marrow–derived macrophages (lower panel). (B) Real-time PCR comparison of various isoforms of adenylate cyclases, beta adrenergic receptor, Gαs transcript variant 1, and Gαi1. The *p* values are shown below the abbreviation of the genes analyzed. Abbreviations: ADCY, adenylate cyclase; ADRB, adrenergic receptor beta; GNAS, alpha subunit of the stimulatory G protein of adenylate cyclase (transcript variant 1); GNAI, homo sapiens guanine nucleotide binding protein (G protein), alpha inhibiting activity polypeptide 1 [*n* = 2 per sample (WT or Cor 1–expressing cells) and three independent RT-PCR runs]. Data are expressed as fold difference relative to wild type. ADCY2, 4, 5, 8, 9, ADRB1, and ADRB3 were below detection values.
(TIF)

Figure S9 **Stimulus-dependent Gα association in neuronal N1E-115 cells.** N1E-115 cells were left untreated or stimulated with isoproterenol (10 µM, min), lysed, and Gα molecules immunoprecipitated as described, followed by separation by SDS-PAGE and immunoblotting using anti–coronin 1 (left) or anti-Gα antibodies (right).
(TIF)

Figure S10 **Coronin 1–dependent activation of cAMP production in the presence and absence of pertussis toxin.** Primary hippocampal neuron cultures from wild-type or coronin 1–deficient brains were stimulated with isoproterenol (5 µM) for 15 min in the presence or absence of pertussis toxin (0.2 mg/ml) and processed for cAMP analysis.
(TIF)

Figure S11 **Expression and localization of the coronin 1 quadruple mutant.** (A) Mel JuSo cells expressing either wild-type coronin 1, the quadruple mutant (Arg-69, Lys-20, Lys-355, and Glu-102 to alanines), or the unstable V134M coronin 1 mutant as C-terminal HA-tagged constructs. Cells were lysed and total proteins separated by SDS-PAGE followed by immunoblotting for coronin 1 and actin. (B) Mel JuSo cells were transfected with the indicated constructs with methanol and stained with anti–coronin 1 antibodies followed by Alexa Fluor 488 and observed by confocal laser scanning microscopy.
(TIF)

Figure S12 **Cortical LTP in the presence and absence of coronin 1 and rescue by 2 h of preincubation with 8-Br-cAMP.** (A–C) Examination of Cortico-LA associative long-term plasticity in wild-type (A) or coronin 1–deficient slices (B) as well as following 8-Br-cAMP pre-incubation in coronin 1–deficient slices. (C, Left panels) Time course of EPSC amplitude. (Right panels) EPSC amplitude distribution before and after the pairing application. Note that following 8-Br-cAMP, pairing successfully induces synaptic plasticity (LTD, 75% of the slice, LTP 25% of the slice), a situation not observed in control coronin 1–deficient slices (in B). (D) Mean CS+-induced freezing during acquisition of cued fear conditioning in wild-type (WT; *n* = 20) and coronin 1 −/− (*n* = 21) mice after intracranial amygdala infusion of 8-Br-cAMP. Main effect genotype×CS trials, F(5,195) = 2.83, *p*<0.01; main effect genotype, F(1,39) = 0.055, *p*>0.05; main effect CS trials, F(5,195) = 66.20, *p*<0.0001, two-way RMANOVA. Bonferroni post hoc analysis revealed no genotype differences.
(TIF)

Figure S13 **Proposed model for the role of coronin 1 in modulation of the cAMP/PKA pathway.** In coronin 1–expressing cells, cell surface stimulation results in the assembly of coronin 1 with Gαs followed by an increase in cAMP production, PKA activation, CREB phosphorylation, and induction of LTP in neurons.
(TIFF)

Movie S1 **Isoproterenol-stimulated cAMP production in coronin 1–expressing Mel JuSo cells.** Cells were stimulated with isoproterenol (5 µM) at the time point of 25 s. The experimental details and data analysis are described in Materials and Methods.
(MOV)

Movie S2 **Isoproterenol-stimulated cAMP production in Mel JuSo control cells.** Cells were stimulated with isoproterenol (5 µM) at the time point of 25 s. The experimental details and data analysis are described in Materials and Methods.
(MOV)

Movie S3 **Isoproterenol-stimulated cAMP production in Latrunculin B treated (4 µM, 30 min) coronin 1–expressing Mel JuSo cells.** The cells were stimulated with isoproterenol (5 µM) at the time point of 25 s. The experimental details and data analysis are described in Materials and Methods.
(MOV)

Movie S4 **Isoproterenol-stimulated cAMP production in Latrunculin B treated (4 µM, 30 min) Mel JuSo control cells.** The cells were stimulated with isoproterenol (5 µM) at the time point of 25 s. The experimental details and data analysis are described in Materials and Methods.
(MOV)

Table S1 **Statistical analysis.**
(XLSX)

Table S2 **Metabolic testing of the patient.**
(PDF)

Table S3 **Coronin 1 mutants and cAMP production.** Wild-type coronin 1 or the mutant molecules indicated were expressed in Mel JuSo cells, subjected to a 2 h serum starvation followed by stimulation with isoproterenol (10 µM for 5 min) and by cAMP analysis as described in Materials and Methods.
(DOC)

Table S4 **RT-PCR primer list.**
(XLSX)

Text S1 **Supporting materials and methods.**
(DOCX)

## Abbreviations

ADR: beta adrenergic receptor
cAMP: cyclic adenosine mono phosphate
EPAC: exchange protein activated by cAMP
EPSC: excitatory post synaptic current
FRET: fluorescence resonance energy transfer
GAD67: glutamic acid decarboxylase 67
G*α*i: G alpha inhibitory
G*α*s: G alpha stimulatory
GPCR: G protein coupled receptor
ICUE3: indicator of cAMP using EPAC
IPSC: inhibitory post synaptic current
LTP: long term potentiation
CREB: cAMP response element binding protein
MRI: magnetic resonance imaging
PKA: Protein Kinase A
PPR: paired pulse ratio
vGAT: vesicular GABA transporter
vGLUT: vesicular glutamate transporter
