# Supplementary figures and images for: Coronin 1 Regulates Cognition and Behavior through Modulation of cAMP/Protein Kinase A Signaling
Source: PLoS Biol. 2014 Mar 25;12(3):e1001820. doi: 10.1371/journal.pbio.1001820 (PMC3965382; doi:10.1371/journal.pbio.1001820)

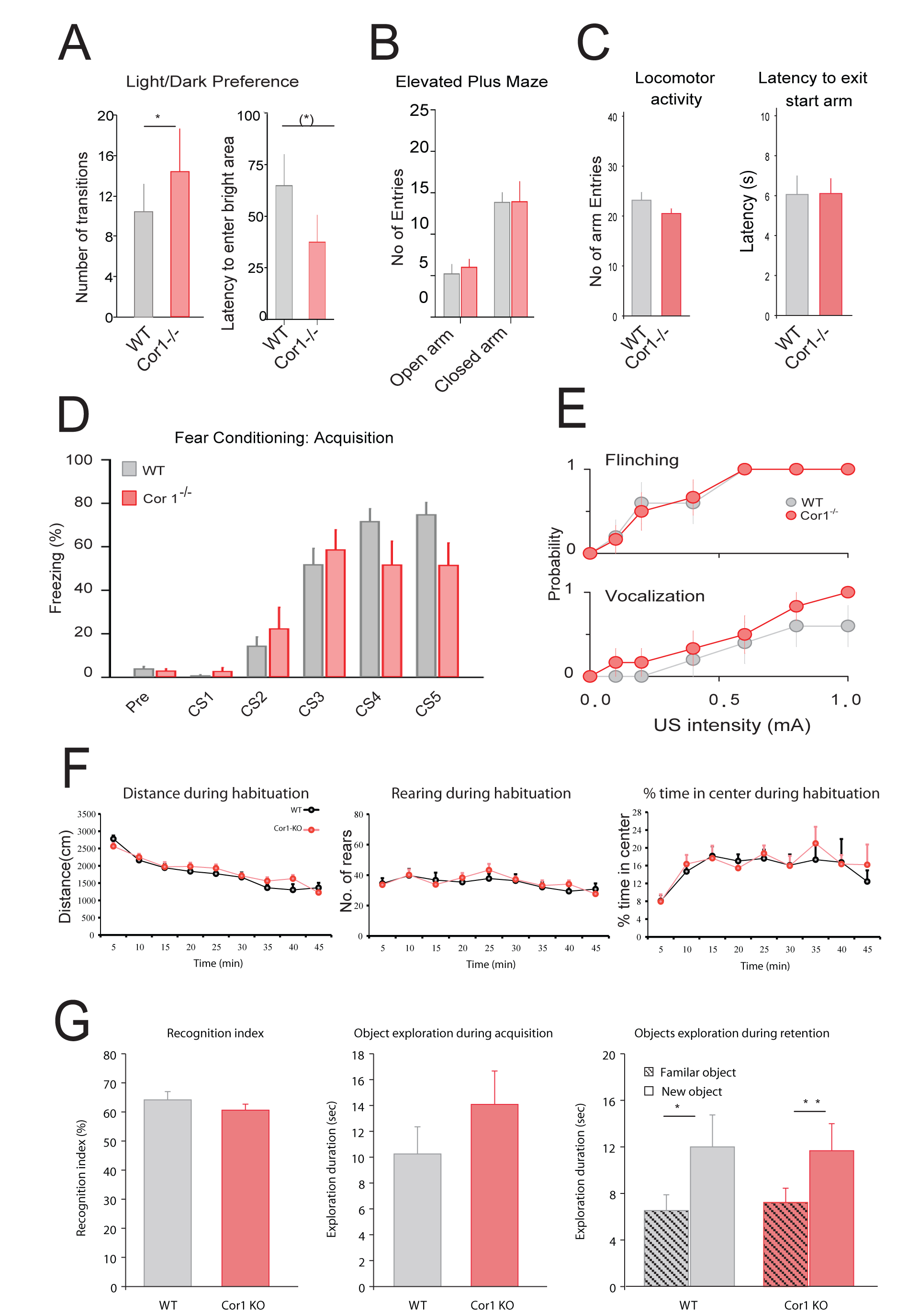

Supplement: Figure S1 — Behavioral phenotyping of wild-type and coronin 1–deficient mice. (A) Light versus dark preference. Number of transitions between the compartments (n = 14 WT, 16 cor 1 −/− mice). P<0.05, Student's t test. Latency to enter bright compartment (n = 14 WT, 16 cor 1 −/− mice). P = 0.091, Mann–Whitney U test. (B) Elevated plus maze, number of arm entries. No significant difference between wild-type and cor 1 −/− mice (n = 16 WT, 15 cor 1 −/− mice). (C) Locomotor activity. Motor function was tested in wild-type and cor 1 −/− animals using a Y-maze (n = 12 WT, 12 cor 1 −/− mice). (D) Mean CS+-induced freezing during acquisition of cued fear conditioning in wild-type (WT; n = 12) and cor 1 −/− (n = 11) mice. (E) US-sensitivity. Foot-shock–induced flinching behavior and vocalization were not different between coronin 1 −/− (n = 6) and wild-type (n = 5) mice (p>0.05, Student's t test for both behaviors). (F, G) Open field and novel object recognition (n = 12 WT, 12 cor 1 −/− mice). *p<0.05, Student's t test. **p<0.01, Student's t test. See Table S1 for additional statistics. (TIF) [file pbio.1001820.s001.tif]

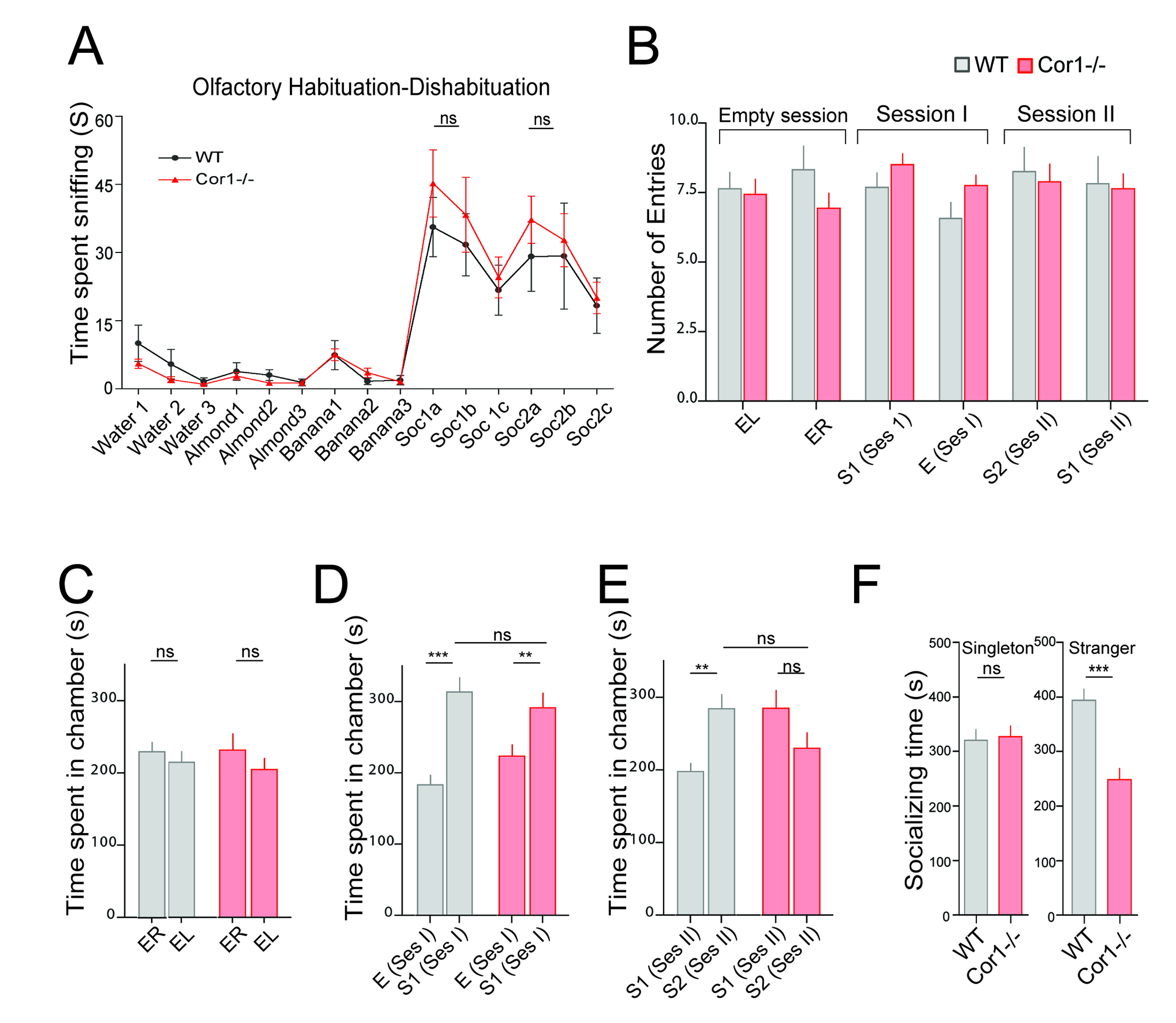

Supplement: Figure S2 — Social interaction of wild-type and coronin 1–deficient mice. (A) Similar olfactory function of coronin 1 −/− mice and wild-type mice as analyzed using various cues (soc, social cue) (ns; RM-ANOVA). n = 10 WT and 10 cor 1 −/−. (B–E) Three-chamber socialization assay. (B) Number of entries in the chambers indicated; E, empty; L, left; R, right; S1 and S2, stranger mouse 1 and 2; ses I, session I (E versus S1); ses II, session II (S2 versus S1). (C–E) Time spent in chamber for the cages in habituation session (C), session I (D), and session II (E). Coronin 1 −/− mice show a reduced sociability in session 1 and a reduced social novelty in session 2 relative to wild-type mice. RMANOVA, **p<0.01 and ***p<0.001. n = 16 WT and 16 Cor 1 −/−. (F) Reduced social interaction of cor 1 −/− mice as assessed by modified Paylors partition method. Student's t test, p<0.0001. n = 13 WT, 14 cor 1 −/− (for Singletons) 13 WT and 16 Cor 1−/− (for stranger). See Table S1 for additional statistics. (TIF) [file pbio.1001820.s002.tif]

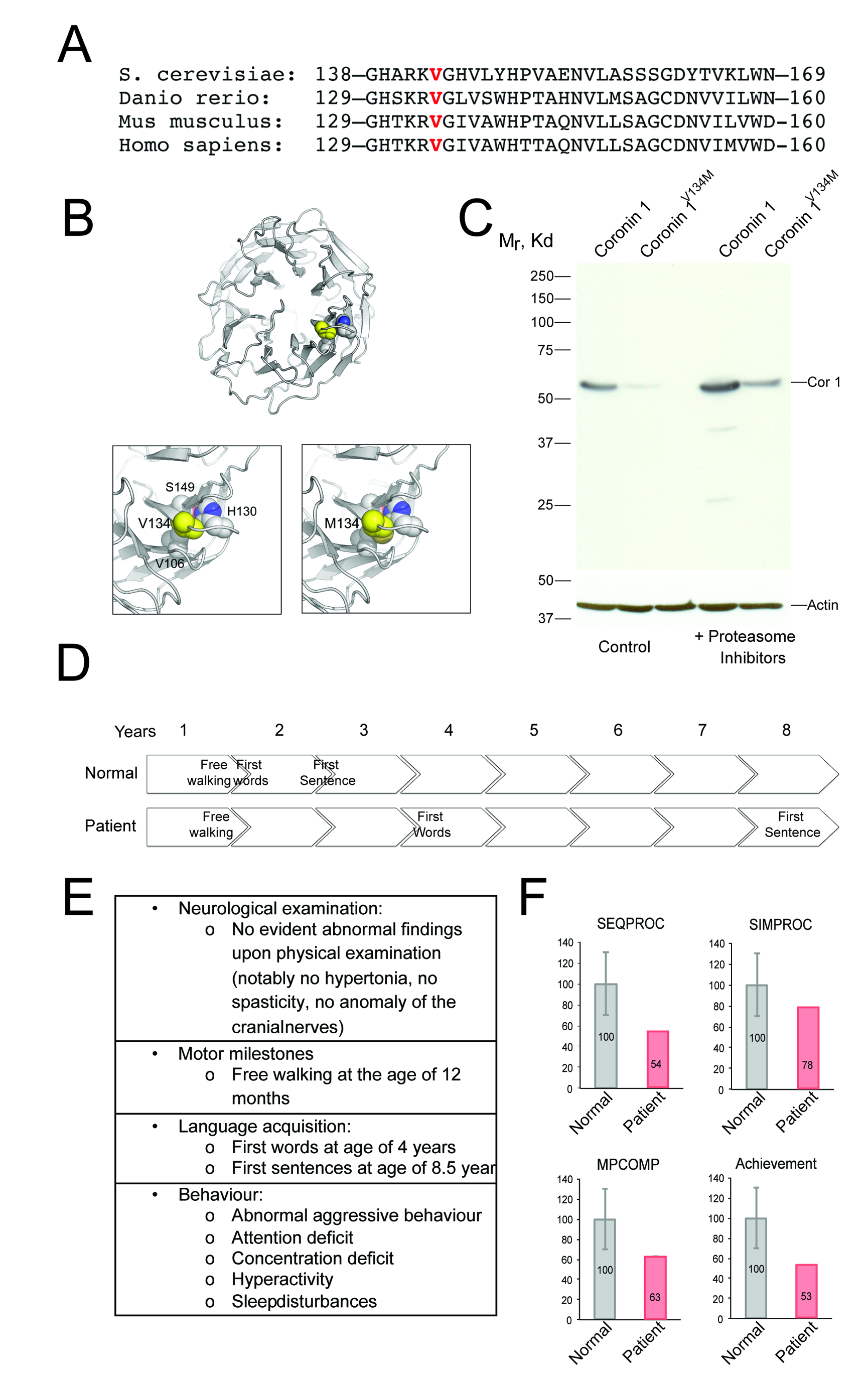

Supplement: Figure S3 — A point mutation in human coronin 1 causes severe cognitive disabilities. (A) Alignment of yeast and zebrafish coronin and murine and human coronin 1. (B) Location of the valine-methionine mutation at position 134. (C) Cell lysates from HEK293 cells transfected with cDNA encoding either wild-type coronin 1, coronin 1V134M or left untransfected (lane 3), were analyzed by SDS-PAGE and immunoblotting using anti–coronin 1 antiserum (upper panels) or anti-actin antibodies (lower panels). Left, untreated. Right, proteasome-inhibitor-treated. (D) Motor skills and language acquisition. (E) Neurological characterization of the patient. (F) Scores on the K-ABC for the patient at 8 y and 8 mo of age compared to normal controls. (TIF) [file pbio.1001820.s003.tif]

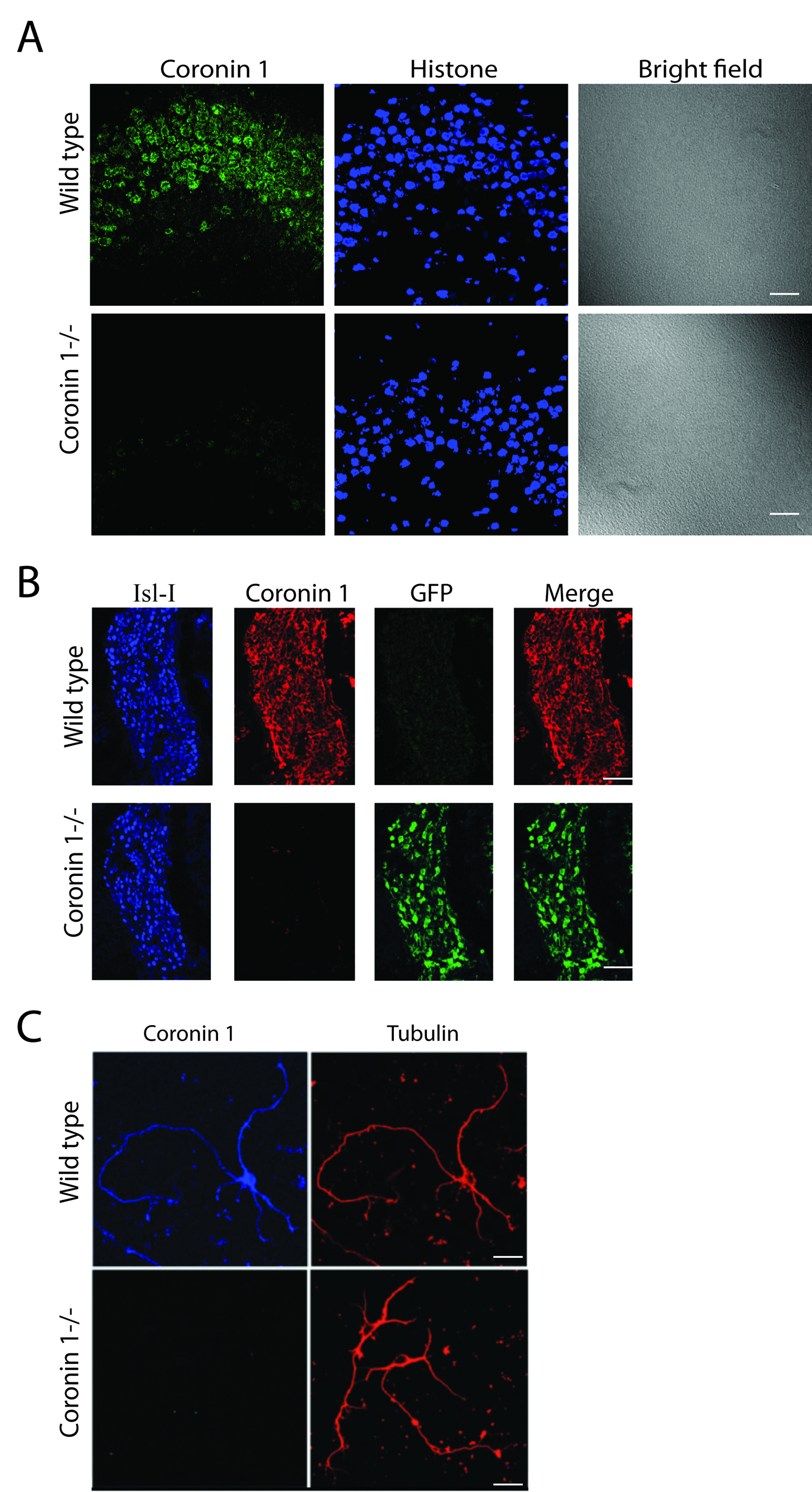

Supplement: Figure S4 — Localization of coronin 1 by immunofluorescence analysis in wild-type and coronin 1–deficient sections and cultured neurons. (A–C) Characterization of coronin 1 expression in wild-type and coronin 1–deficient hippocampi (A), dorsal root ganglion (B), and primary hippocampal cultures (C) using coronin 1 rabbit anti-serum followed by Alexa Fluor 488 (A), Alexa Fluor-568 (B), or Alexa Fluor 647 (C) as well as the other markers indicated. Scale bar, 30 µm (A), 50 µm (B), and 20 µm (C). (TIF) [file pbio.1001820.s004.tif]

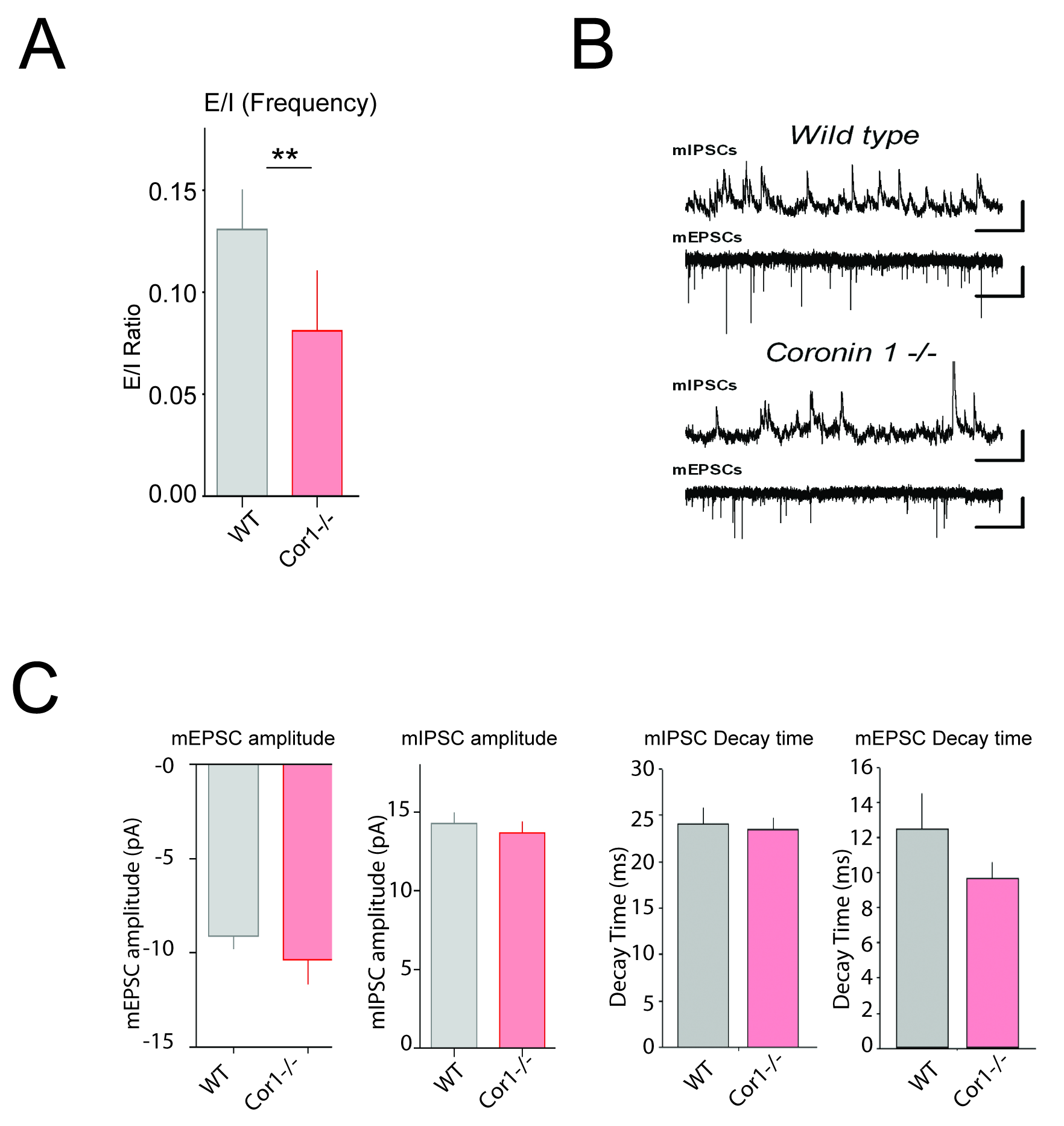

Supplement: Figure S5 — Altered mEPSC frequencies in the absence of coronin 1. (A) E/I ratio [n = 13 cells for wild-type (grey) and n = 12 cells for coronin 1–deficient (red)]. p<0.01, Mann–Whitney U test. (B) Excitatory and inhibitory transmission in the hippocampus CA1 pyramidal neurons. Scale bars, 20 pA and 0.5 s (mIPSC) and 5 sec (mEPSC). (C) Miniature EPSC and IPSC's amplitudes and decay times recorded from wild-type and coronin 1–deficient hippocampus; n = 13 wild-type, 12 Cor1 −/−, p>0.05 (mEPSC), p>0.05 (mIPSC), Student's t test, see Table S1. (TIF) [file pbio.1001820.s005.tif]

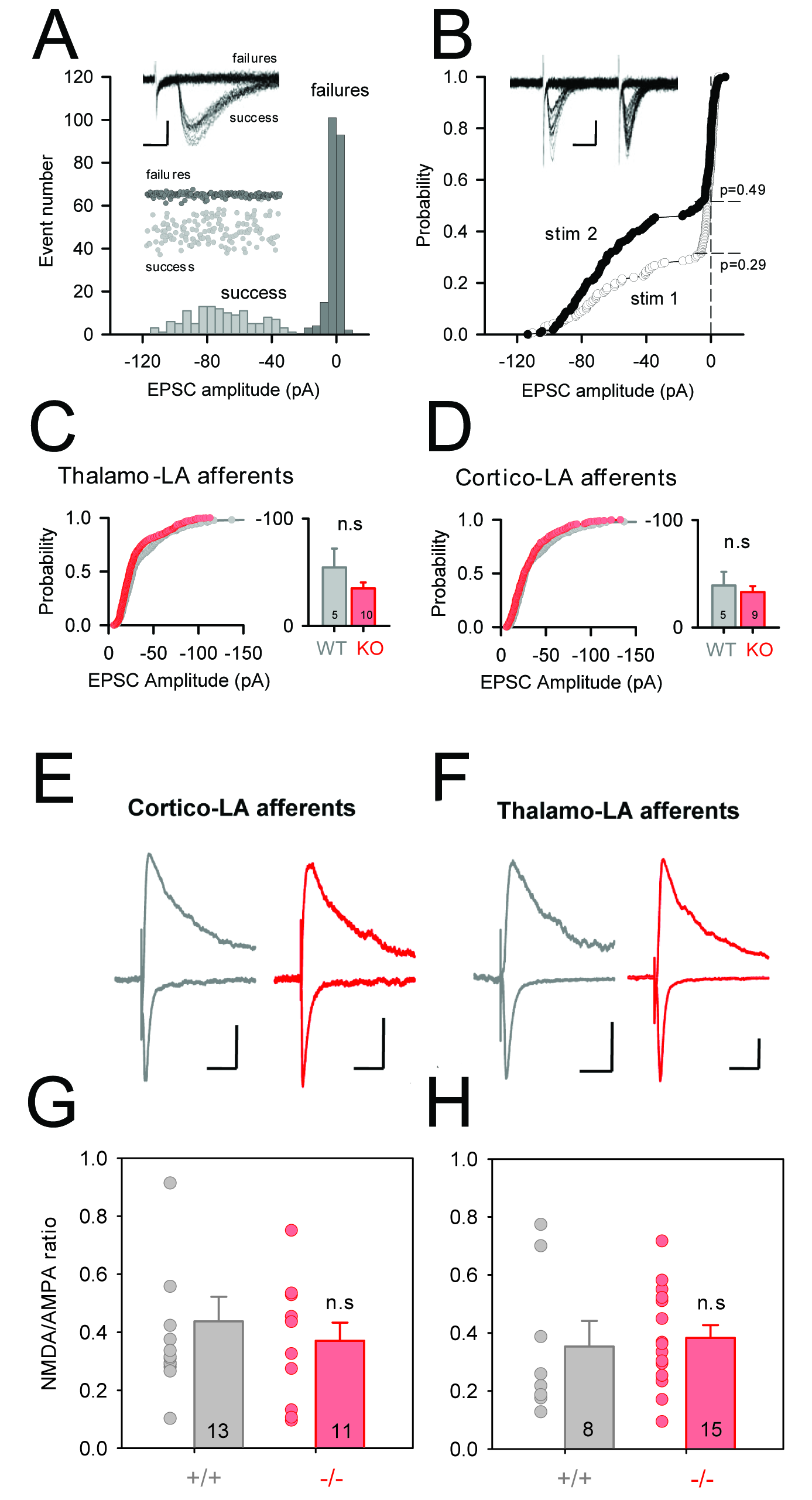

Supplement: Figure S6 — Electrophysiology of thalamo- and cortico-LA afferents in wild-type and coronin 1–deficient mice. (A) Minimal stimulation of afferent axons typically results in well-separated EPSC responses (successes) and failures. Scale bars, 30 pA and 5 ms. (B) Consistent with the double stimulation of a single axon, the release probability (success rate) is increased when a second stimulation occurs at a short interval (50 ms). Scale bars, 30 pA and 15 ms. (C, D) Summary graphs illustrating normal amplitude of thalamo- and cortico-LA minimal responses in coronin 1–deficient mice (n numbers are indicated on bar graphs; n.s., not significant), p>0.05, Student's t test. (E) The cortico-LA AMPA receptor-mediated component of synaptic transmission was quantified at −70 mV (peak amplitude). The NMDA receptor-mediated component of synaptic transmission was quantified at +50 mV (amplitude at 100 ms after stimulation). Scale bars, 50 pA and 50 ms. (G) Cortico-LA NMDA/AMPA ratios did not differ between wild-type (WT) and coronin 1–deficient (Cor1 −/−) mice. Scale bars, 50 pA and 50 ms. n, numbers are indicated on bar graphs, p>0.05, Student's t test. (F, H) Same as (E, G) for thalamo-LA synapses, p>0.05, Student's t test. (TIF) [file pbio.1001820.s006.tif]

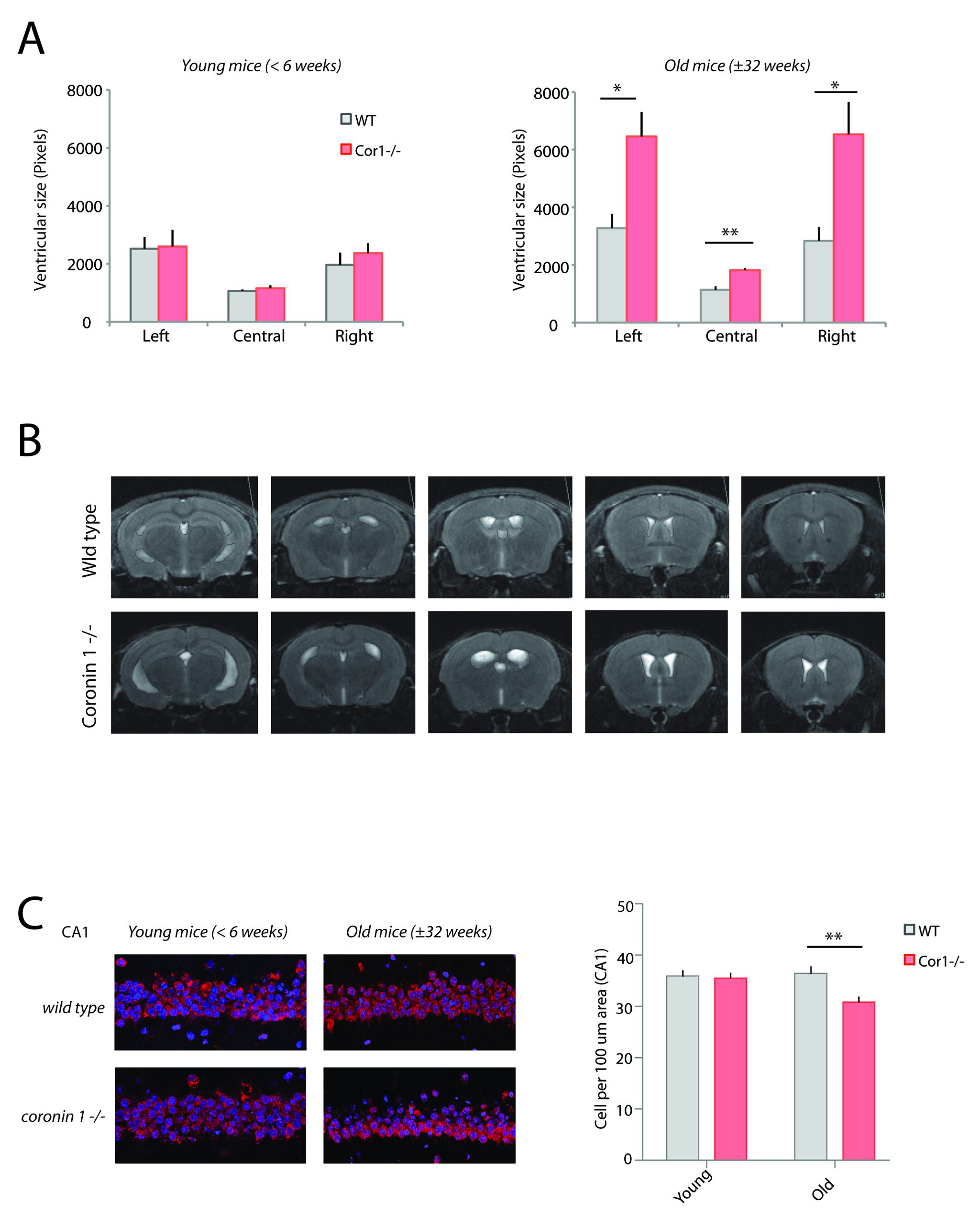

Supplement: Figure S7 — MRI analysis of brain ventricles and histology in wild-type and coronin 1–deficient animals. (A) Ventricle sizes in the presence and absence of coronin 1 as analyzed by MRI of mice aged either less than 6 wk or ∼32 wk. n = 4. Shown are mean ventricular size (left, right, and middle) ± SEM. p<0.05, 0.01, and 0.05 for left, central, and right ventricle, respectively (Student's t test). (B) Sequential MRI imaging of a representative wild-type and coronin 1–deficient mouse. (C, Left panels) CA1 hippocampal regions of age-matched male wild-type and coronin 1–deficient mice stained with neurotrace red and Dapi and imaged using a confocal microscope (Zeiss LSM 700). Scale bar,20 µm. (Right panels) Quantitation of the neurotrace red-positive cell numbers in the CA1 hippocampal region (n = 3 mice per genotype). The data (mean ± SEM) are represented as the numbers of neurons per 100-µm linear length of medial CA1 (n = 10–12 regions from three different mice in each group). p<0.01 (Student's t test), see also Table S1. (TIF) [file pbio.1001820.s007.tif]

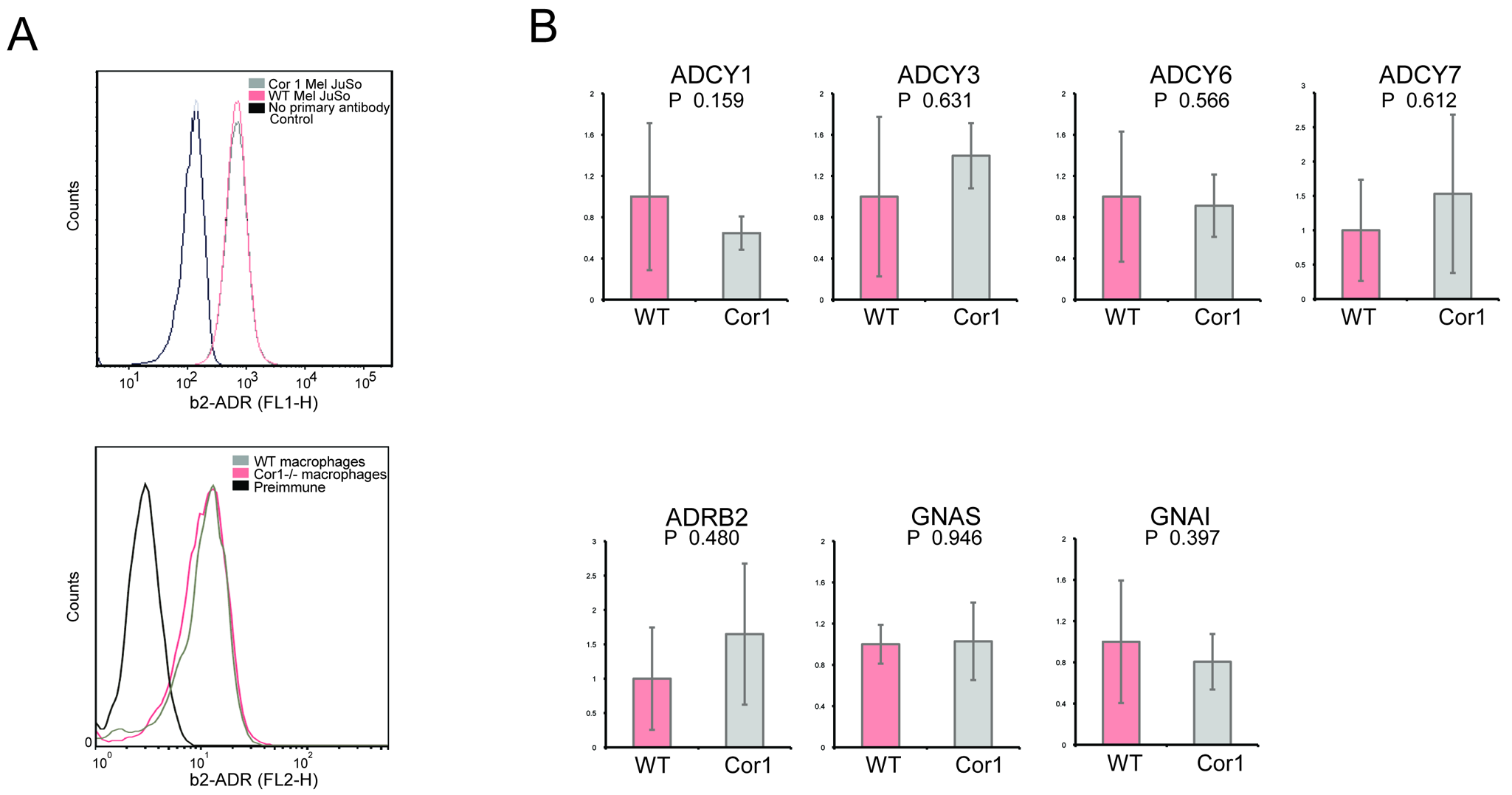

Supplement: Figure S8 — Effect of coronin 1 on expression of components of the beta-adrenergic receptor signaling pathway. (A) FACS analysis of surface expression of β2-adrenergic receptor in wild-type and coronin 1–expressing Mel JuSo cells (upper panel) and bone-marrow–derived macrophages (lower panel). (B) Real-time PCR comparison of various isoforms of adenylate cyclases, beta adrenergic receptor, Gαs transcript variant 1, and Gαi1. The p values are shown below the abbreviation of the genes analyzed. Abbreviations: ADCY, adenylate cyclase; ADRB, adrenergic receptor beta; GNAS, alpha subunit of the stimulatory G protein of adenylate cyclase (transcript variant 1); GNAI, homo sapiens guanine nucleotide binding protein (G protein), alpha inhibiting activity polypeptide 1 [n = 2 per sample (WT or Cor 1–expressing cells) and three independent RT-PCR runs]. Data are expressed as fold difference relative to wild type. ADCY2, 4, 5, 8, 9, ADRB1, and ADRB3 were below detection values. (TIF) [file pbio.1001820.s008.tif]

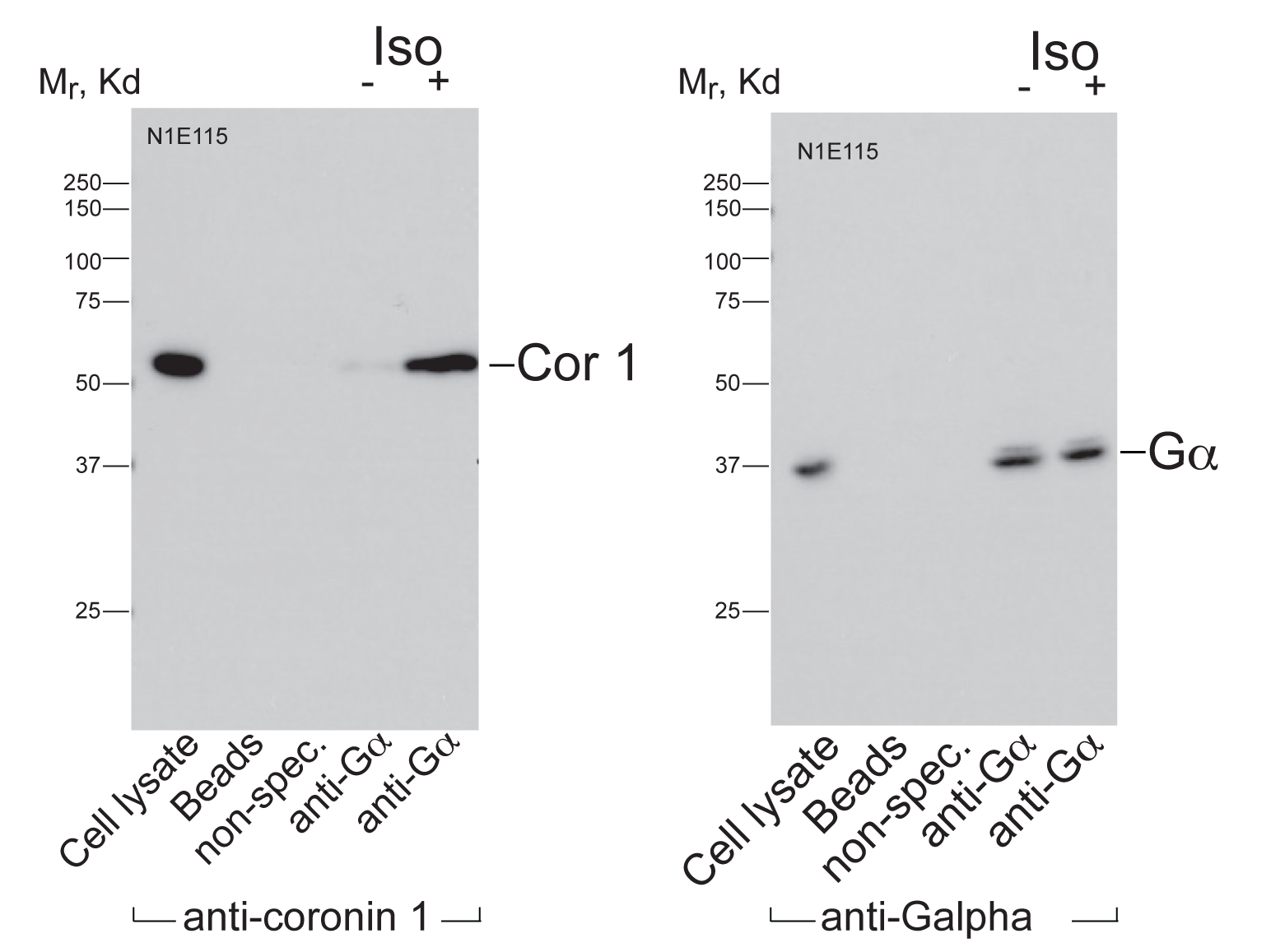

Supplement: Figure S9 — Stimulus-dependent Gα association in neuronal N1E-115 cells. N1E-115 cells were left untreated or stimulated with isoproterenol (10 µM, min), lysed, and Gα molecules immunoprecipitated as described, followed by separation by SDS-PAGE and immunoblotting using anti–coronin 1 (left) or anti-Gα antibodies (right). (TIF) [file pbio.1001820.s009.tif]

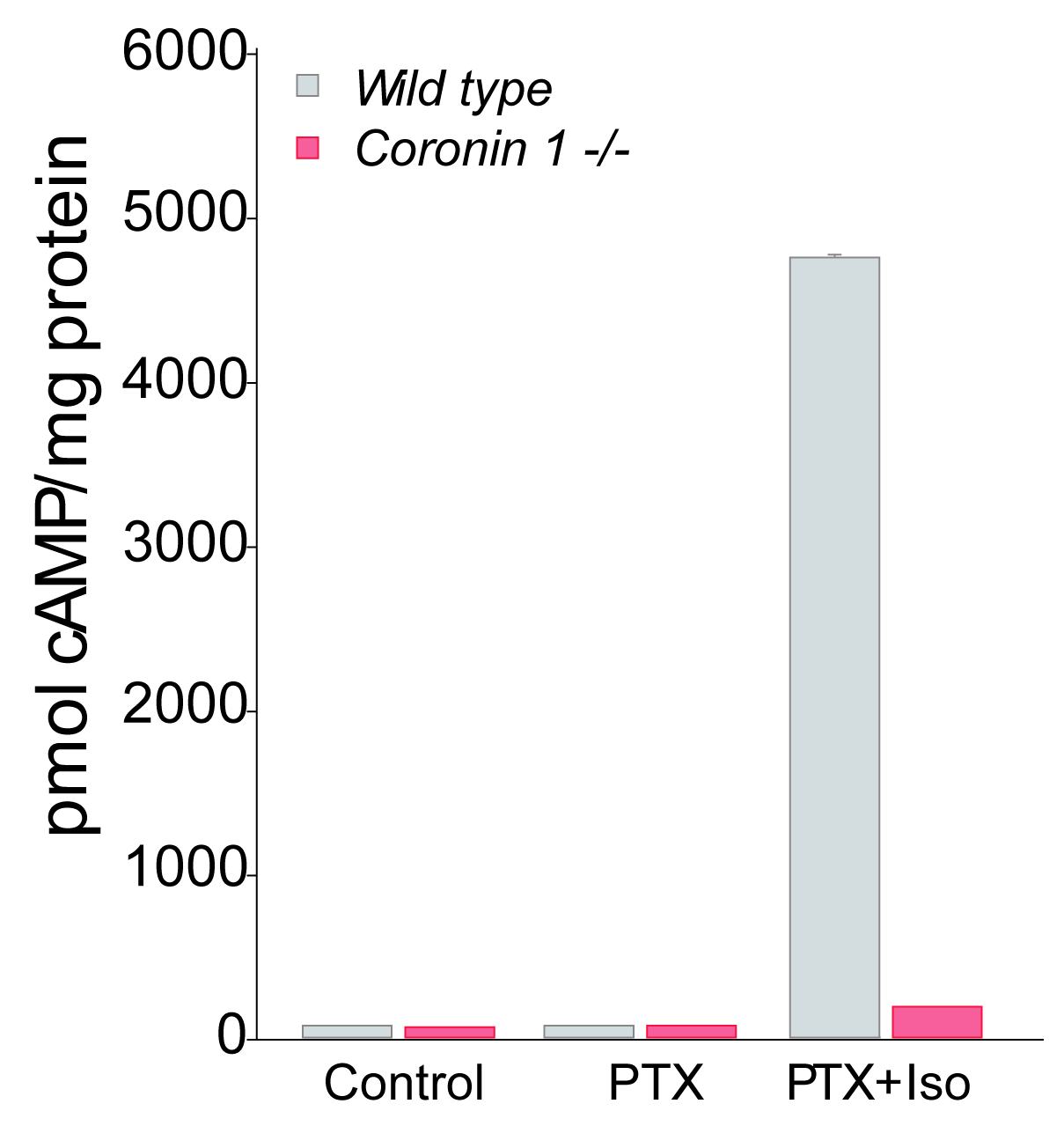

Supplement: Figure S10 — Coronin 1–dependent activation of cAMP production in the presence and absence of pertussis toxin. Primary hippocampal neuron cultures from wild-type or coronin 1–deficient brains were stimulated with isoproterenol (5 µM) for 15 min in the presence or absence of pertussis toxin (0.2 mg/ml) and processed for cAMP analysis. (TIF) [file pbio.1001820.s010.tif]

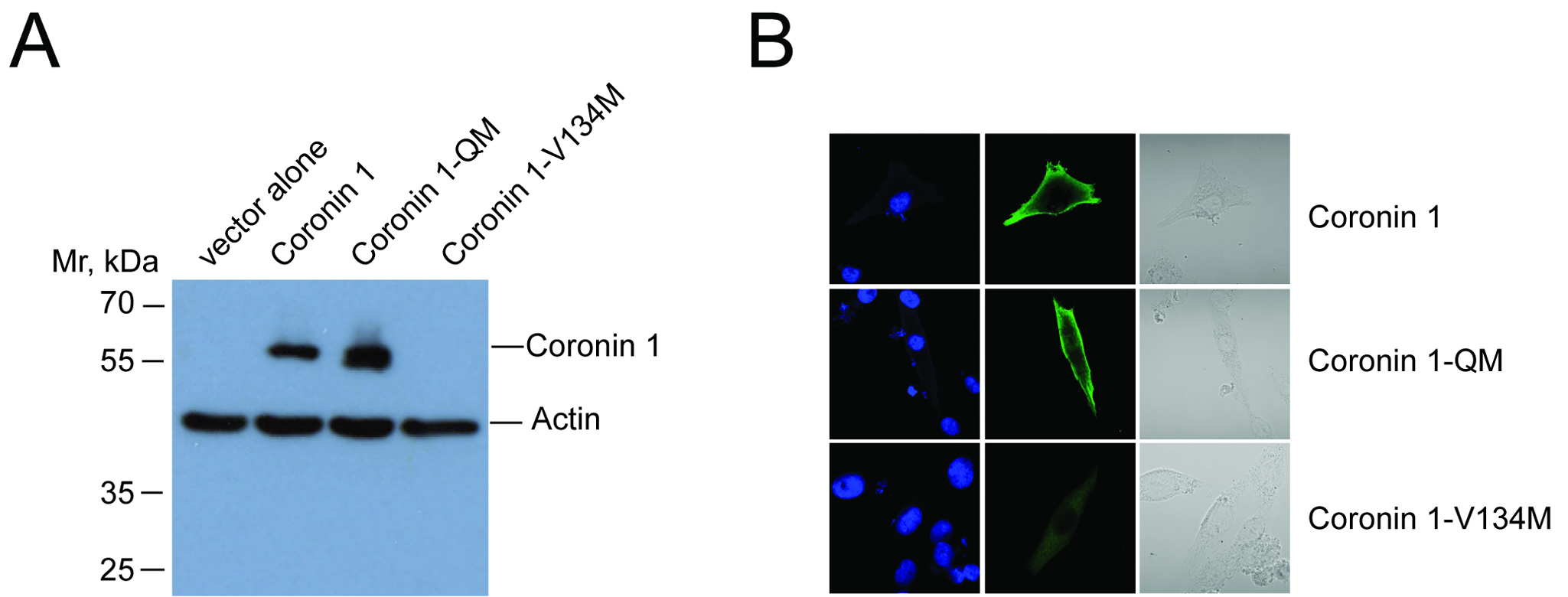

Supplement: Figure S11 — Expression and localization of the coronin 1 quadruple mutant. (A) Mel JuSo cells expressing either wild-type coronin 1, the quadruple mutant (Arg-69, Lys-20, Lys-355, and Glu-102 to alanines), or the unstable V134M coronin 1 mutant as C-terminal HA-tagged constructs. Cells were lysed and total proteins separated by SDS-PAGE followed by immunoblotting for coronin 1 and actin. (B) Mel JuSo cells were transfected with the indicated constructs with methanol and stained with anti–coronin 1 antibodies followed by Alexa Fluor 488 and observed by confocal laser scanning microscopy. (TIF) [file pbio.1001820.s011.tif]

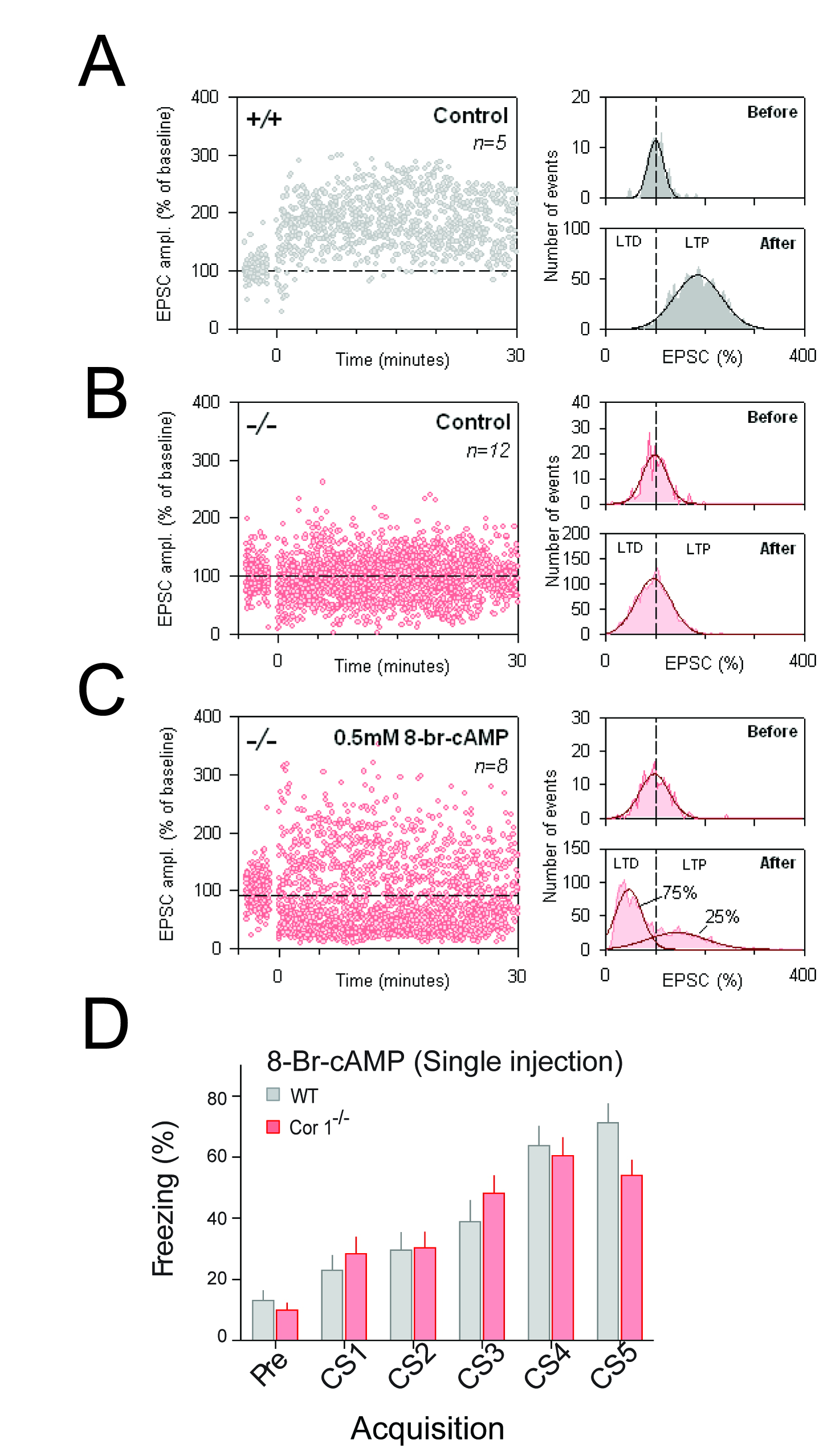

Supplement: Figure S12 — Cortical LTP in the presence and absence of coronin 1 and rescue by 2 h of preincubation with 8-Br-cAMP. (A–C) Examination of Cortico-LA associative long-term plasticity in wild-type (A) or coronin 1–deficient slices (B) as well as following 8-Br-cAMP pre-incubation in coronin 1–deficient slices. (C, Left panels) Time course of EPSC amplitude. (Right panels) EPSC amplitude distribution before and after the pairing application. Note that following 8-Br-cAMP, pairing successfully induces synaptic plasticity (LTD, 75% of the slice, LTP 25% of the slice), a situation not observed in control coronin 1–deficient slices (in B). (D) Mean CS+-induced freezing during acquisition of cued fear conditioning in wild-type (WT; n = 20) and coronin 1 −/− (n = 21) mice after intracranial amygdala infusion of 8-Br-cAMP. Main effect genotype×CS trials, F(5,195) = 2.83, p<0.01; main effect genotype, F(1,39) = 0.055, p>0.05; main effect CS trials, F(5,195) = 66.20, p<0.0001, two-way RMANOVA. Bonferroni post hoc analysis revealed no genotype differences. (TIF) [file pbio.1001820.s012.tif]

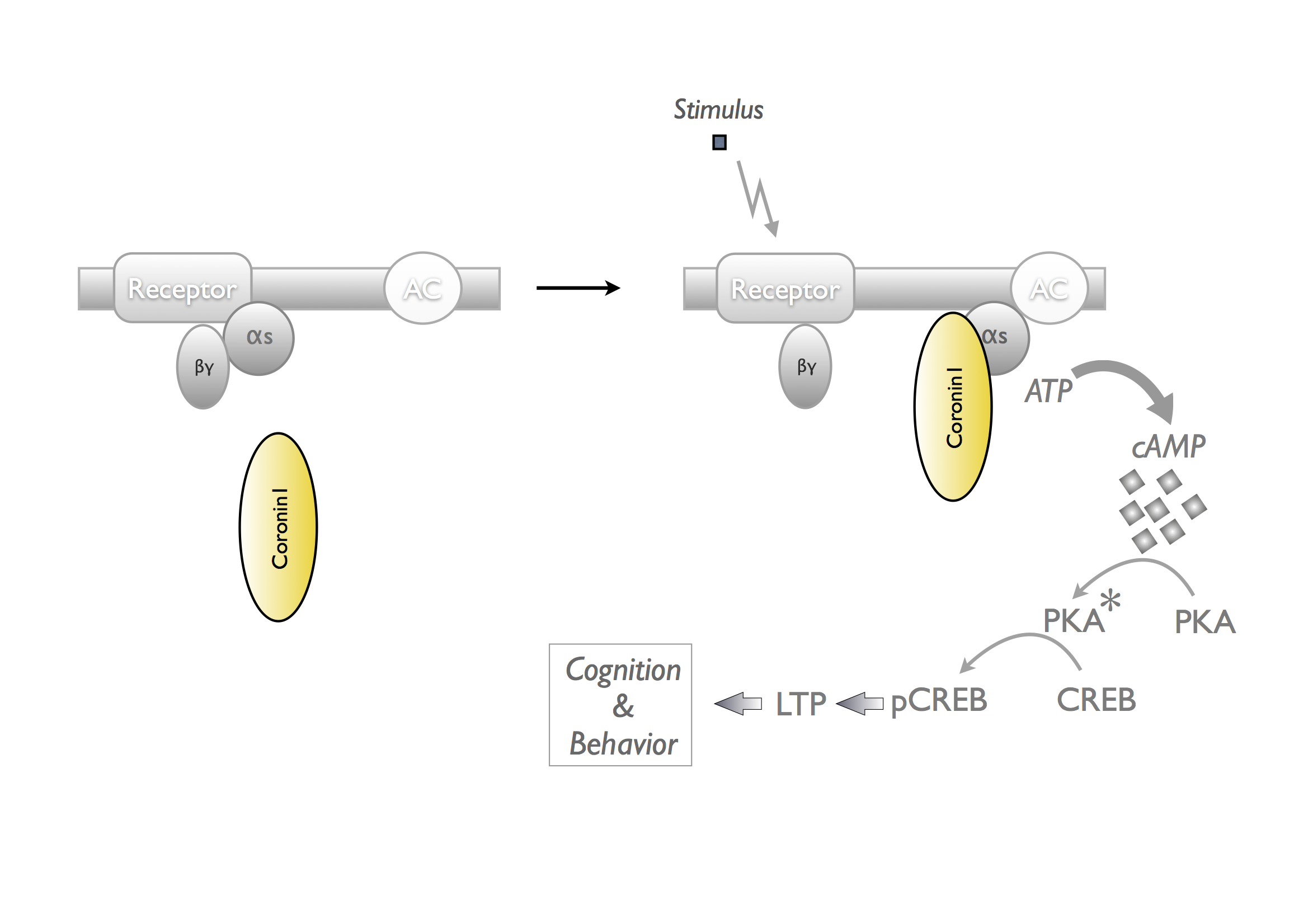

Supplement: Figure S13 — Proposed model for the role of coronin 1 in modulation of the cAMP/PKA pathway. In coronin 1–expressing cells, cell surface stimulation results in the assembly of coronin 1 with Gαs followed by an increase in cAMP production, PKA activation, CREB phosphorylation, and induction of LTP in neurons. (TIFF) [file pbio.1001820.s013.tif]
