# Supplementary material for: Coronin 1 Regulates Cognition and Behavior through Modulation of cAMP/Protein Kinase A Signaling
Source: PLoS Biol. 2014 Mar 25;12(3):e1001820. doi: 10.1371/journal.pbio.1001820 (PMC3965382; doi:10.1371/journal.pbio.1001820)
Supplement: Table S2 — Metabolic testing of the patient. (PDF) [file pbio.1001820.s019.pdf]

Table S2

Metabolic Testing:

• plasma and urine amino and organic acid analysis,	Normal Range
• urine oligosaccharide and mucopolysaccharides,	Normal Range
• urine purines and pyrim-idines	Normal Range
• leukocyte enzymes	Normal Range
• screening for mitochondri-al disorders (analysis of lac-tate in blood and cerebro-spinal fluid, lactate to py-ruvate ratio, hydroxybutyr-ate to acetic acid ratio, mo-lecular study of mitochon-drial DNA)	No abberancies
• ammonia in blood	Normal Range
• Vitamine B12 and Folate	Normal Range
• cerebrospinal fluid	Normal
• fatty acid and very long chain fatty acids	Normal
• serum copper levels	Normal Range
• carnitine	No deficiency
