# Supplementary material for: Coronin 1 Regulates Cognition and Behavior through Modulation of cAMP/Protein Kinase A Signaling
Source: PLoS Biol. 2014 Mar 25;12(3):e1001820. doi: 10.1371/journal.pbio.1001820 (PMC3965382; doi:10.1371/journal.pbio.1001820)
Supplement: Table S3 — Coronin 1 mutants and cAMP production. Wild-type coronin 1 or the mutant molecules indicated were expressed in Mel JuSo cells, subjected to a 2 h serum starvation followed by stimulation with isoproterenol (10 µM for 5 min) and by cAMP analysis as described in Materials and Methods. (DOC) [file pbio.1001820.s020.doc]

**Table S3: Coronin 1 mutants and cAMP production**

| **Coronin 1 mutant** | **cAMP production** |
| --- | --- |
|  |  |
| Wild type | ++++++ |
| R317A/L62A/K355A | ++++++ |
| K132A/R69A/R317A | ++++++ |
| K20A/T131A/R133A/E102A | +++++ |
| K20A/R69A/K355A | ++ |
| K20A/R69A/E102A | ++ |
| L62A/T131A/R133A/R69A | ++++++ |
| R69A/T131A/R133A/ K355A | +++ |
