# Supplementary material for: Coronin 1 Regulates Cognition and Behavior through Modulation of cAMP/Protein Kinase A Signaling
Source: PLoS Biol. 2014 Mar 25;12(3):e1001820. doi: 10.1371/journal.pbio.1001820 (PMC3965382; doi:10.1371/journal.pbio.1001820)
Supplement: Text S1 — Supporting materials and methods. (DOCX) [file pbio.1001820.s022.docx]

**Supporting Information:**

**Essential Role for Coronin 1 in Neuronal cAMP/Protein kinase A signaling Regulates Cognition and Behavior**

Rajesh Jayachandran*, Xiaolong Liu*, Somdeb BoseDasgupta*, Philipp Mueller*, Chun-Lei Zhang^🟁^, Despina Moshous**^¶^**, Vera Studer^+^, Jacques Schneider^∇^, Christel Genoud^, Catherine Fossoud^◆^, Frédéric Gambino^🟁^, Malik Khelfaoui^🟁^, Christian Müller^#^, Deborah Bartholdi^∇✚^, Helene Rossez*, Michael Stiess*, Xander Houbaert^🟁^, Rolf Jaussi^^, Daniel Frey, Richard A. Kammerer^^, Xavier Deupi^±^ , Jean-Pierre de Villartay**^¶^**, Andreas Lüthi^#^, Yann Humeau^🟁^ and Jean Pieters*

**Supporting Materials and Methods:**

**Behavioral analysis.** *Resident-intruder test.* Mice (male, 16-23 weeks) were individually housed for 4 weeks (“residents”). In parallel, another mouse of similar body weight were “group housed” (four per cage) for a similar length of time (“intruder”). To initiate the analysis, the intruder was transferred into the resident cage and the interaction behavior monitored for 10 minutes as described [[1](#_ENREF_1),[2](#_ENREF_2)]. The mouse that attacked the opponent mouse first and/or showed dominant posture (biting, chasing, aggressive grooming and scratching of the opponent) was considered the winner, while a mouse that was attacked by the opponent or showed submissive posture (freezing, crouching, inactivity) was considered the loser. If the mice displayed no signs of dominant or submissive behavior during the entire 10 minutes duration, the outcome was considered a draw (n=37 independent trials). For calculation of attack latency, the time of attack was scored. Mice which did not attack the intruder were given an attack latency score of 600 seconds [[3](#_ENREF_3)].

*Tube displacement test*. An age matched coronin 1-deficient and wild type mouse were first placed in the opposite side chambers with their nose pointing towards the tube opening. They were released simultaneously and allowed to enter the tube by opening the carton plug. The mice were monitored for push/pull behavior until one of the mice fully retreated from the tube with the first mouse to exit being scored as ‘‘loser,’’ (i.e., the less aggressive/dominant of the two) and the other mouse the winner. If none of the mice exit the tube within 10 min or if the mice were to cross each other, the experiment was terminated and scored as a draw. The percentage of mice of a genotype that wins over the opponent was scored and plotted (n=33 trials, from three independent studies).

*Dark versus light.* These compartments were interconnected to each other via a smaller central compartment of dimension 10 cm x10 cm in the middle by means of an opening located at floor level. Light intensity was maintained at 8 lux in the black compartment and 400 lux in the white compartment and both the compartments were covered with a red Plexiglas lid to avoid any external interference. Mice (males, 17-22 weeks) were placed in the central compartment and allowed to explore freely for a duration of 10 minutes. The cages were cleaned in between every experiment with water and ethanol. The amount of time spent in the light area as well as the number of transitions were monitored. A transition was scored when all the four paws of the mice were inside one of the chambers. (n=14 for wild type, n=16 for coronin 1-deficient).

*Rotarod test***:** Mice (males, 16-17 weeks) were given three testing trials on a rotating rod (Bioseb, Chaville, France) during which the rotation speed accelerated from 4 to 40 rpm in 5 min. Trials were separated by 15 min interval. The average latency was used as index of motor coordination performance (n=12 per group).

*Hot plate test:* The mice (males, 18-19 weeks) were placed into a glass cylinder on a hot plate adjusted to 52°C (Bioseb). Two trials separated with 5-10 min interval were carried out. For the first trial, the latency of the first reaction (licking, flinches, little leaps) was recorded, with a maximum of 30 sec. For the second trial, in addition to the first reaction, the latency to jump was also recorded. A 3 min cut-off was used for the second trial (n=12 per group).

*Tail flick test:* The apparatus consisted of a shutter-controlled lamp as a heat source (Bioseb). The temperature of the heat bundle was controlled by fixing the focus and sensitivity of the system. Three consecutive trials with an interval of about 2-3 min were performed at different sites of the tail. For each trial, the tail of the animal (males, 18-19 weeks) was placed under the heat source. The time taken by the animal to flick its tail was recorded (cut off 20s; n=12 per group).

*Formalin test:* Mice (males, 19-20 weeks) were placed in a restrainer and received a subcutaneous injection of formalin (4%, 20 μl) into the plantar aspect of the left hind paw. Immediately after formalin injection, the mice were placed in Plexiglas cylinder and the duration of licking/biting behavior was recorded for 1 min each 5 minutes period up to 60 min after formalin injection[[4](#_ENREF_4),[5](#_ENREF_5)] (n=12 per group).

*Beam walking:* The apparatus used was a 2 cm diameter and 110 cm long wooden beam, elevated 50 cm above the ground. The beam is subdivided into 11 segments allowing one to estimate rapidly the distance crossed by the mouse (males, 16-17 weeks). The first segment at one extremity of the beam is used as starting point during testing trials. A goal box (12 X 12 X 14 cm) is fixed at the other extremity of the beam. Animals were first habituated to the goal box for 1 min. Animals were submitted to 3 training trials during which they were placed at different points of the beam the head directed to the goal box and allowed to walk the corresponding distance to enter the goal box. After training, animals were submitted to 3 testing trials during which they were placed at the extremity of the beam opposite to the goal box and allowed to walk the beam distance and enter the goal box. The latency to enter the goal box and the number of slips (when one or both hind paws slips laterally from the beam) were measured (n=12 per group).

*Open Field****:*** The open-fields (44.3 x 44.3 x 16.8 sm) were placed in a room homogeneously illuminated at 70 Lux at the level of each open field. Animals (wildtype and coronin 1-deficient males, 15-16 weeks, n=12) were first habituated to the open-field for 45 min. followed by analysis of distance moved in the open field, number of rears and percentage of time spent in the center of the open field.

*Novel Object Recognition****:*** The object recognition task was performed in automated open fields which were placed in a room homogeneously illuminated at 70 Lux. The objects to be discriminated were a glass marble (2.5 cm diameter) and a plastic dice (2 cm). Animals (males, 15-16 weeks; n=12) were first habituated to the open-field for 45 min. The next day, they were submitted to a 10-minutes acquisition trial during which they were placed in the open-field in presence of an object A (marble or dice). The time the animal took to explore the object A (when the animal’s snout was directed towards the object at a distance ≤ 1 cm) was manually recorded. A 10-minutes retention trial was performed 3 h later. During this trial, the object A and another object B were placed in the open-field, and the times tA and tB the animal takes to explore the two objects were recorded. A recognition index (RI) is defined as (tB / (tA + tB)) x100.

*Grooming analysis.* Wild type and an age and sex matched coronin 1-deficient mouse were placed separately into the compartments and allowed to explore freely for a duration of 5 minutes. They were subsequently assessed for 10 min for the amount of time they spent self-grooming as well as the number of times they performed self-grooming. The cages were cleaned between every analysis with water and ethanol. n=13 mice per genotype (males, 20-30 weeks) per group from three independent experiments.

*Elevated plus maze test.* For this study, mice were given one 10-min trial on the plus maze, which has two closed arms, with walls 40 cm in height, and two open arms intersecting in the center area (9.5 cm x 9.5 cm). The maze was elevated 97 cm from the floor, and the arms were 30 cm long. Wild type or coronin 1-deficient mice (males, aged 12-20 weeks,) were placed on the center and allowed to freely explore the maze. Mice were monitored for the time spent in open arms, closed arms and center of the maze and the number of entries into the open and closed arms were noted. Mice were considered to have entered one of the arms when all the four paws were placed inside the arm under consideration. Percent open arm time was calculated as 100 x (time spent on the open arms/(time in the open arms + time in the closed arms)). Percent open arm entries was calculated using the same formula, but using the measure for entries. The maze was wiped with water and ethanol after every session. N=16 wild type and 15 coronin 1-deficient mice. All the mice under study were shifted to the experimental room at least 30 minutes prior to the initiation of the study.

*Olfactory Habituation-Dishabituation analysis* The test mouse was gently transferred into a new standard cage with bedding. A cotton swab was inserted from the top of the gridded plate of the cage such that its cotton tip was placed at a height of approximately 8 cm from the bottom of the bedding. The mice was habituated in this setting for at least 30 minutes after which a series of various olfactory cues were provided by replacing the cotton swab with cotton swabs soaked in various olfactory cues including water, almond (100μl of benzaldehyde, 1:3000 in water), banana (100μl of isoamylacetate, 1:1500 in water) and social cues. Social cues were obtained by repeatedly swabbing cotton swabs into the bedding material of an unrelated mice cage that had been left unchanged for at least 5 days with the resident mice. Water, non-social cues, and social cues were introduced sequentially to the test mice for 3 minute duration each with every cue repeated three times. Cues were retrieved from the cage after each session and the inter-session interval was 1 min. The sniffing behavior toward cue-containing cotton swabs was measured. A mouse was considered sniffing if it placed its nostrils within 2 cm from the tip of the cotton swab and the total duration of sniffing was monitored in parallel with a stop clock by an observer. N=10 per genotype, 13-20 weeks in age.

*Three Chamber Social Interaction Assay.* The test consisted of three sessions; habituation session, sociability session and social novelty session. To initiate the habituation session (empty session), the test mouse (male, 11-19 weeks) whose socialization behavior was analyzed was placed in the middle chamber with the dividers opened to allow it to explore the middle and side chambers for ten minutes. As the animal moved through each opening, the time of entry into that compartment was noted. Additionally, the time spent in each chamber and the transitions between chambers were monitored. After this ten-minute habituation period, the test mouse was gently guided to the middle chamber and the dividers closed. To initiate the test for sociability, an unfamiliar adult C57BL/6 male mouse serving the purpose as social stimulus 1 (stranger 1, S1, male, aged 12-24 weeks) was placed inside one of the stranger cages with the other stranger cage remaining empty and the dividers raised, allowing the test mice to move freely once again throughout all three chambers for a 10-minute test session. The time spent and number of entries into the chamber containing the stranger mouse 1 (S1) or the chamber containing the empty stranger cage (abbreviated E_R_ or E_L_) was monitored for 10 minutes and scored as described [[6](#_ENREF_6)]. In addition, each test mouse was analyzed for the duration of sniffs directed towards the wire cages containing the stranger mouse and sniffs directed towards the empty wire cage with the nose less than 2 cm of the stranger cage, to measure exploration time. Preference for social novelty was analyzed in a subsequent session (session II) for a duration of 10 minutes after leading the test mouse to the middle chamber as in the prior session. The original stranger mouse (S1) remained in its stranger cage. An unfamiliar novel mouse serving the purpose as social stimulus 2, (stranger 2 (S2), male, aged 12-24 weeks) was placed in the stranger cage on the opposite side. Identical measures as previously described were scored (time spent in each chamber, entries between chambers and time spent sniffing each wire cage). Stranger 1 and stranger 2 animals originated from different home cages and had never been in physical contact with the subject mice or each other prior to the analysis. The stranger mice were habituated to sit in the stranger cage for a duration of 10 minutes every day prior to the experimental day for at least 10 days. The empty side versus the stranger 1 side and like wise the stranger 1 versus the stranger 2 sides were swapped throughout the experiment to minimize any artifact that could arise due to a side preference even though no such side preference were observed for both the genotypes. Socialization was also analyzed using a modified Paylor test as described [[2](#_ENREF_2)]. In brief, a rectangular, black Plexiglas box of dimension 80 cm x 30 cm was divided into two equal compartments using a transparent perforated Plexiglas partition placed in the middle (multiple perforations, diameter 1 cm) and covered with a red Plexiglas lid. For assessment as a singleton mouse (‘singleton’), mice (males, 12-16 weeks) were placed inside one compartment of the chamber with the other chamber left empty for a duration of 5 minutes to habituate. Subsequently, the mice were monitored for a duration of 10 minutes and the amount of time spent in the inner one third of the chamber (close to the partition) was scored as socialization time. The cages were cleaned in between with water and ethanol. n=13-16 mice per group, three independent experiments. For assessment in the presence of a stranger (‘stranger’), wild type or coronin 1-deficient mice (males, 12-16 weeks) were placed in one of the compartments, along with another (novel, wild type C57BL6) mouse in the opposite compartment. Monitoring and scoring was done as above.

*Vocalization.* Both the compartments were covered with a red Plexiglas lid to avoid any external interference. The test mouse (wild type or coronin 1-deficient male, ± 14-34 weeks), was placed in one of the compartment and the number of calls emitted in both audible and ultrasonic frequency over a duration of 10 minutes were recorded using an ultra sound gate 116 device (Avisoft bioacoustics, Berlin) and processed using Avisoft-SAS Labpro Software and Ravenpro 1.4 (Cornell Lab of Ornithology). The cages were cleaned between the experiments with ethanol (n=15 wild type,13 coronin 1-deficient mice, two independent experiments).

*Fear conditioning.* The conditioning and retrieval boxes and the floor were cleaned with 70% ethanol or 1% acetic acid before and after each session, respectively. To score freezing behavior an automatic infrared beam detection system placed on the bottom of the experimental chambers (Coulbourn Instruments, Allentown, PA) was used. The animals were considered to be freezing if no movement was detected for 2 s. On day 1, mice were submitted to a habituation session in context A, in which they received 4 presentations of the CS^+^ and the CS^–^ (total CS duration: 30 s, consisting of 50 ms pips repeated at 0.9 Hz, 2 ms rise and fall, pip frequency: 7.5 kHz or 3 kHz, 80 dB). Discriminative fear conditioning was performed the same day by pairing the CS^+^ with a US (1 s foot-shock, 0.6 mA, 5 CS^+^-US pairings; inter-trial interval: 20-180 s). The onset of the US coincided with the offset of the CS^+^. The CS^–^ was presented after each CS^+^/US association but was never reinforced (5 CS^–^ presentations, inter-trial interval: 20-180 s). The frequencies used for CS^+^ and CS^–^ were counterbalanced across animals. On day 2, conditioned mice were submitted to a retrieval test in context B during which they received 4 presentations of the CS^–^ and the CS^+^, respectively.

**Study subjects and neurological examination***.* We reviewed the charts of a patient (currently 11 years old) with an identified homozygous missense mutation in coronin 1 in order to evaluate the neurological development. The identification of the coronin 1 mutation is described in detail elsewhere [[7](#_ENREF_7)]. Karyotyping revealed a normal chromosomal number and composition with no major anomaly. Comparative Gene Hybridization (CGH) array analysis using DNA extracted from fibroblast culture was carried out with the Affymetrix CytoScan HD platform. This array platform includes 2.67 million markers for copy number analysis, approximately 750’000 SNP probes and 1.9 million non-polymorphic oligo probes for whole-genome coverage. The data were analyzed with the Affymetrix Chromosome Analysis Suite software (ChAS version 32.1, genome build GRCh37/hg19) and compared to an internal reference constructed from more than 200 hap map samples. Data were analyzed for deletions or duplications up to 50 kb and for smaller aberration overlapping known disease genes. Detected variants were compared to polymorphic variants displayed in the DGV database. The analysis did not reveal any known disease causing copy number variants in this patient. Written informed consent from the patients’ parents was obtained for genetic and functional analysis. This retrospective study was conducted according to the French ethical legislation. The patient was repeatedly evaluated by child neurologists, child psychiatrists, geneticists, psychologists and speech therapists. In addition, electroneuromyography at the age of 2 years showed normal sensory and motor nerve conduction. Ophthalmologic examination (including funduscopy, electroretinogram and visual-evoked potential) was also normal. Auditory-evoked potential was normal besides a slight transmission hypoacusis due to chronic otitis that could not per se explain the delay in language acquisition. His cognitive level was estimated with the help of standardized test, including the Raven's Progressive Matrices [[8](#_ENREF_8)] the K-ABC [[9](#_ENREF_9)] and the NEMI 2 [[10](#_ENREF_10)].

**Mutagenesis and cell transfection.** Val 134 on mouse coronin 1 was mutated by using specific primers which cause a GTT to ATG mutation (forward primer: CCA CAC CAA GAG GAT GGG CAT CGT GGC CTG; reverse primer: CAG GCC ACG ATG CCC ATC CTC TTG GTG TGG), with pCB6_mouse coronin 1 as a template [[11](#_ENREF_11)]. Following PCR amplification of the plasmid (Pfu turbo polymerase (Agilent)) the methylated parental plasmid was digested with DpnI and the mixture transformed into *E. coli* DH5a and plated onto ampicillin containing LB-agar plates. Plasmids were prepared using the Plasmid miniprep kit (Zymo Research) and confirmed by sequencing (Microsynth). For transfection, HEK293 cells were transfected with Fugene 6, and expression analyzed in the presence and absence of proteasome inhibitors lactacystein (ALLN (20 μM), epoxomicin (2 μM) and clasto-lactacystin-β-lactone (2 μM) by immunoblotting.

For site-directed mutagenesis of residues K20, R69, E102, and K355 to alanine, PCR reactions were carried out using specific mutagenic primers as indicated in the table below with the mutated nucleotides in red.

| Mutation | Primers name | Primer Sequence |
| --- | --- | --- |
| K20A | Cor1K20Af  Cor1K20Ar | GGACAGCCAGCCGCGGCTGACCAGTGC  GCACTGGTCAGCCGCGGCTGGCTGTCC |
| R69A | Cor1R69Af  Cor1R69Ar | CCCCTAGGCAAGACTGGAGCAGTAGACAAGAACGTGCCC  GGGCACGTTCTTGTCTACTGCTCCAGTCTTGCCTAGGGG |
| E102A | Cor1E102Af  Cor1E102Ar | GCCAGTGGCTCTGCGGACTGCACAGTTATGG  CCATAACTGTGCAGTCCGCAGAGCCACTGGC |
| K355A | Cor1K355Af  Cor1K355Ar | CCATGACAGTGCCTAGAGCGTCGGACCTGTTCCAGG  CCTGGAACAGGTCCGACGCTCTAGGCACTGTCATGG |

PCR cycling of the plasmid pCB6-Cor1 [[11](#_ENREF_11)] was carried out according to the following protocol: Initial denaturation at 95^o^C for 1 min, 18 cycles of 50 sec denaturation at 95 ^o^C, 50 sec annealing at 60 ^o^C and 6.5 min of extension at 68 ^o^C, followed by final extension at 68 ^o^C for 8 min and finally to 4 ^o^C. The PCR products were thereafter digested with 1 unit of DpnI overnight. Next day 15 μl of PCR product was used to transform ultracompetent DH5alpha cells. Plasmids were prepared and sequenced for each mutation.

**Supporting References**

___________________________

1. Koolhaas JM, Schuurman T, Wiepkema PR (1980) The organization of intraspecific agonistic behaviour in the rat. Prog Neurobiol 15: 247-268.

2. Spencer CM, Alekseyenko O, Serysheva E, Yuva-Paylor LA, Paylor R (2005) Altered anxiety-related and social behaviors in the Fmr1 knockout mouse model of fragile X syndrome. Genes, brain, and behavior 4: 420-430.

3. Kaidanovich-Beilin O, Lipina TV, Takao K, van Eede M, Hattori S, et al. (2009) Abnormalities in brain structure and behavior in GSK-3alpha mutant mice. Molecular brain 2: 35.

4. Sufka KJ, Watson GS, Nothdurft RE, Mogil JS (1998) Scoring the mouse formalin test: validation study. European journal of pain 2: 351-358.

5. Chesler EJ, Ritchie J, Kokayeff A, Lariviere WR, Wilson SG, et al. (2003) Genotype-dependence of gabapentin and pregabalin sensitivity: the pharmacogenetic mediation of analgesia is specific to the type of pain being inhibited. Pain 106: 325-335.

6. Moy SS, Nadler JJ, Young NB, Perez A, Holloway LP, et al. (2007) Mouse behavioral tasks relevant to autism: phenotypes of 10 inbred strains. Behavioural brain research 176: 4-20.

7. Moshous D, Martin E, Carpentier W, Lim A, Callebaut I, et al. (2013) Whole-exome sequencing identifies Coronin-1A deficiency in 3 siblings with immunodeficiency and EBV-associated B-cell lymphoproliferation. The Journal of allergy and clinical immunology.

8. Raven J, Raven JC, Court JH (2003 updated 2004) Manual for Raven's Progressive Matrices and Vocabulary Scales. . San Antonio, TX: Harcourt Assessment.

9. Kaufman AS, Kaufman NL (2004) Kaufman Assessment Battery for Children Second Edition.: Circle Pines, MN: American Guidance Service.

10. Cognet G ( 2006) NEMI 2 : nouvelle echelle metrique de l’intelligence 2. ; ECPA LEdCdPa, editor.

11. Gatfield J, Albrecht I, Zanolari B, Steinmetz MO, Pieters J (2005) Association of the Leukocyte Plasma Membrane with the Actin Cytoskeleton through Coiled Coil-mediated Trimeric Coronin 1 Molecules. Mol Biol Cell 16: 2786-2798.
